# Intensive Microalgal Cultivation and Tertiary Phosphorus Recovery from Wastewaters via the EcoRecover Process

**DOI:** 10.1021/acs.est.3c10264

**Published:** 2024-04-30

**Authors:** Hannah R. Molitor, Ga-Yeong Kim, Elaine Hartnett, Benjamin Gincley, Md Mahbubul Alam, Jianan Feng, Nickolas M. Avila, Autumn Fisher, Mahdi Hodaei, Yalin Li, Kevin McGraw, Roland D. Cusick, Ian M. Bradley, Ameet J. Pinto, Jeremy S. Guest

**Affiliations:** † Department of Civil & Environmental Engineering, Newmark Civil Engineering Laboratory, University of Illinois Urbana−Champaign, Urbana, Illinois 61801, United States; ‡ Clearas Water Recovery, Inc., Missoula, Montana 59808, United States; § School of Civil and Environmental Engineering, Georgia Institute of Technology, Atlanta, Georgia 30332, United States; ∥ Department of Civil, Structural and Environmental Engineering, University at Buffalo, The State University of New York, Buffalo, New York 14260, United States; ⊥ Institute for Sustainability, Energy and Environment, University of Illinois Urbana−Champaign, Urbana, Illinois 61801, United States; # Department of Civil and Environmental Engineering, Rutgers, The State University of New Jersey, Piscataway, New Jersey 08854, United States; ∇ Research and Education in Energy, Environmental and Water (RENEW) Institute, University at Buffalo, The State University of New York, Buffalo, New York 14260, United States

**Keywords:** photobioreactors, membrane bioreactor, nutrient recovery, circular bioeconomy, microalgal-bacterial community, microalgae, biopolymer

## Abstract

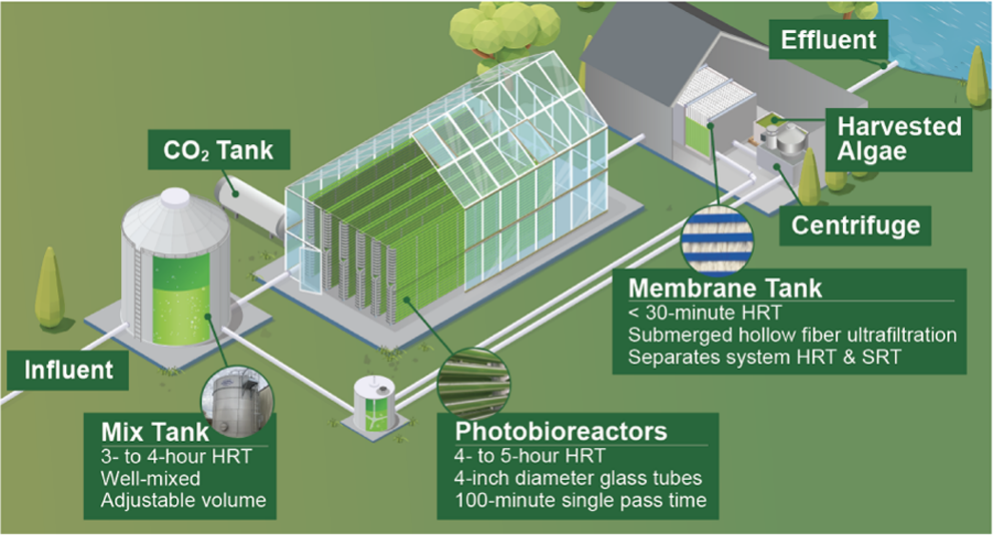

Mixed community microalgal wastewater treatment technologies have the potential to advance the limits of technology for biological nutrient recovery while producing a renewable carbon feedstock, but a deeper understanding of their performance is required for system optimization and control. In this study, we characterized the performance of a 568 m^3^·day^–1^ Clearas EcoRecover system for tertiary phosphorus removal (and recovery as biomass) at an operating water resource recovery facility (WRRF). The process consists of a (dark) mix tank, photobioreactors (PBRs), and a membrane tank with ultrafiltration membranes for the separation of hydraulic and solids residence times. Through continuous online monitoring, long-term on-site monitoring, and on-site batch experiments, we demonstrate (i) the importance of carbohydrate storage in PBRs to support phosphorus uptake under dark conditions in the mix tank and (ii) the potential for polyphosphate accumulation in the mixed algal communities. Over a 3-month winter period with limited outside influences (e.g., no major upstream process changes), the effluent total phosphorus (TP) concentration was 0.03 ± 0.03 mg-P·L^–1^ (0.01 ± 0.02 mg-P·L^–1^ orthophosphate). Core microbial community taxa included *Chlorella spp.*, *Scenedesmus spp.*, and *Monoraphidium spp.*, and key indicators of stable performance included near-neutral pH, sufficient alkalinity, and a diel rhythm in dissolved oxygen.

## Abstract 2

This study characterizes mechanisms of phosphorus recovery in the first full-scale installation of the EcoRecover-mixed community microalgal wastewater treatment process.

## 1 Introduction

The United States Environmental Protection Agency estimates that nutrients impair 15–41% of assessed surface water area (including lakes, rivers, estuaries, etc.) in the United States.^1^ Phosphorus, specifically, is the limiting nutrient for harmful algal growth and eutrophication in many freshwater ecosystems.^2^ To protect or restore US waters, states are adopting numeric water quality criteria for nitrogen and phosphorus by identifying impaired waterbodies and adjusting effluent permits for water resource recovery facility (WRRF) to meet waterbody-specific loadings.^3^ As of 2021, eight states had gained state-wide phosphorus criteria for at least one waterbody type, while another 16 states had added numeric criteria for select waterbodies (headwaters, wadeable streams, reservoirs requiring algicide, etc.).^4^ To meet increasingly prevalent and increasingly stringent effluent phosphorus limits to protect natural waterbodies, wastewater treatment plants are in need of effective and cost-efficient technologies that reliably achieve phosphorus removal or recovery.

To date, commercialized tertiary wastewater treatment technologies for phosphorus management have been limited to enhanced biological phosphorus removal (EBPR), chemical polishing, and membranes.^5^ EBPR can be a lower cost among these options but cannot reliably treat below 0.3 mg·L^–1^ total phosphorus.^6^ Though chemical polishing with coagulants (typically aluminum sulfate or ferric chloride) can achieve more stringent effluent limits, significant addition of these chemicals generates large quantities of sludge that are difficult to treat, are expensive to landfill, and that represent a recalcitrant precipitate that make phosphorus recovery challenging.^7^ Additionally, reliably achieving very low phosphorus effluent concentrations (e.g., <0.1 mg-P·L^–1^)^8^ requires coagulant dosing that is significantly higher than predicted by stoichiometric quantities due to numerous side reactions, and the stoichiometric disparity increases substantially as target effluent phosphorus concentrations decrease.^9^ Once precipitated, phosphorus recovery from chemical polishing sludge requires chemical extraction and/or thermal approaches at very high temperatures (1000 to 2000 °C).^10^ As an alternative to bacteria-driven luxury uptake in EBPR and to chemical polishing, microalgae can achieve phosphorus recovery—including organic phosphorus that is otherwise recalcitrant in conventional WRRFs—^11,12^through assimilation into new biomass,^13^ luxury uptake (as polyphosphate),^14^ and surface adsorption.^15^ If algal treatment systems can be engineered to reliably meet effluent nutrient permits, they have the potential to leverage waste phosphorus for CO_2_ fixation, to enable the recovery of phosphorus from harvested biomass, and to serve as a feedstock for the production of renewable bioproducts and biofuels in support of a circular economy.^16^

Phototrophic wastewater treatment processes continue to be developed in both attached growth and suspended growth configurations. Attached growth systems, such as the revolving algal biofilm (RAB)^17^ and the rotating algal biofilm reactor,^18^ grow mixed communities as biofilms and often cycle them between (i) submerged conditions for nutrient uptake and (ii) atmospheric conditions to support gas exchange (including CO_2_ uptake) and exposure to light. Recent characterization of RAB systems across the Midwest U.S. has demonstrated the potential for luxury phosphorus uptake (as polyphosphate) in RAB communities.^19^ Polyphosphate—along with polyhydroxyalkanoates and glycogen—is a key biopolymer in the rhythm of (chemotrophic) EBPR systems, helping polyphosphate-accumulating organisms to balance growth across cycling environmental conditions.^20^ Although it is not apparent that polyphosphate serves to balance cellular metabolism in light-driven wastewater treatment systems (as in EBPR systems), the storage and consumption of carbohydrates has been shown to help balance activity across light-dark cycling of suspended growth algae cultures.^21−23^ This past work, however, focused on diel lighting with laboratory-scale cultures, whereas intensive wastewater treatment systems can cycle algae between light and dark conditions at time scales of minutes or hours (faster than day-night cycles) and will select for their own mixed communities of microorganisms. It is important, therefore, that we continue to elucidate the role of biopolymers, including polyphosphate and storage carbohydrates, in real-world installations of phototrophic wastewater treatment systems.

**Figure 1:**
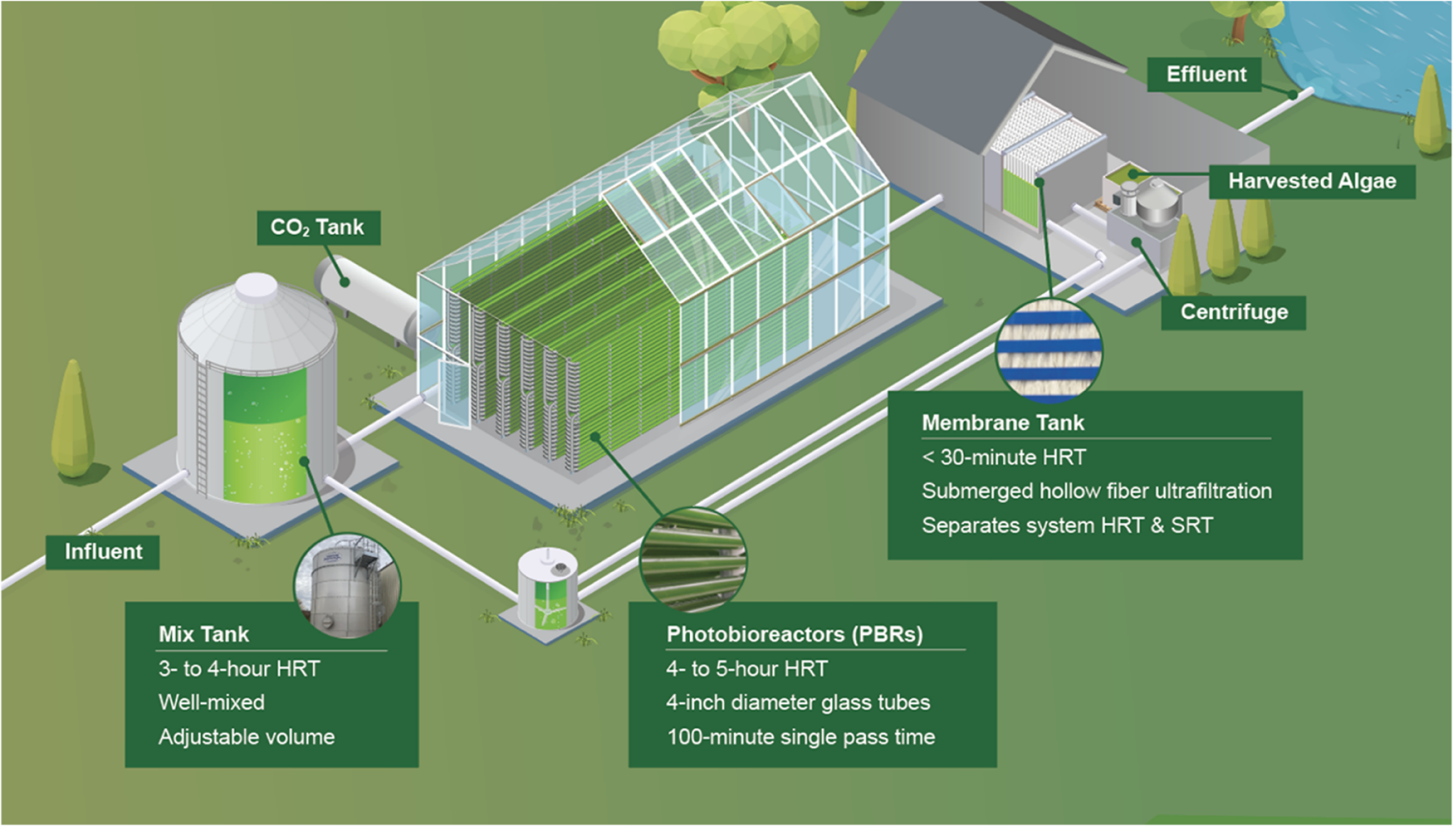
EcoRecover process flow diagram. The mix tank receives secondary effluent and recycled microalgal biomass under dark, nutrient-replete conditions. Inorganic carbon is sparged into the mixed microbial community just prior to the PBRs where the biomass receives light and conditions become phosphorus-limited. Ultrafiltration in the membrane tank (membrane bioreactor) separates the tertiary effluent from the biomass, which is either recycled back to the mix tank or harvested.

The EcoRecover process is an intensive (i.e., high areal productivity, small footprint) tertiary nutrient recovery process which leverages the Advanced Biological Nutrient Recovery (ABNR, Clearas Water Recovery Inc.)^24^ system. The process consists of a dark mix tank, photobioreactors (PBRs), and the separation of the hydraulic retention time (HRT) and solids residence time (SRT) with membranes (Figure 1). The EcoRecover ABNR process was first piloted as a batch system, with minimal monitoring, at the South Davis Sanitary District (South Davis, Utah) beginning in August 2016. In Fall 2021, the first full-scale installation began operation at the Village of Roberts (Wisconsin), with robust monitoring and a design flow of 568 m^3^·day^–1^. As of Fall 2023, a 1,100 m^3^·day^–1^ EcoRecover system has been constructed in Mondovi (Wisconsin) and a 10,600 m^3^·day^–1^ system is operating in Waupun (Wisconsin). To date, however, there is no fundamental understanding of the mechanisms driving phosphorus removal from wastewater (and recovery in biomass) in the EcoRecover process or publicly available data documenting its performance under real-world conditions. Broad adoption of intensive (i.e., high productivity, small footprint) microalgal treatment technologies requires a mechanistic understanding of factors governing phosphorus recovery across unit operations and over 24 h cycles to enable transparent process design and control.

The objective of this work was to elucidate key drivers of phosphorus removal in a full-scale EcoRecover system, including the role of stored carbohydrates in supporting phosphorus uptake under dark conditions and the potential for polyphosphate accumulation. The EcoRecover system was deployed as a tertiary treatment process to achieve effluent total phosphorus concentrations below new water quality-based permit limits of 0.12 mg-P·L^–1^ (monthly) and 0.04 mg-P·L^–1^ (6-month average) for the full forward (design) flow of 568 m^3^·day^–1^ (150,000 gal·day^–1^). Continuous, long-term monitoring was achieved through a network of sensors and analyzers that interfaced with a supervisory control and data acquisition system (SCADA) and were complemented by alternate-day elemental analysis of the solids and twice daily aqueous and total suspended solids (TSS) analyses during weekdays. These long-term monitoring data were supplemented with batch kinetic assays to characterize nutrient and carbohydrate storage dynamics in the mix tank and PBRs. Ultimately, a deeper understanding of the EcoRecover system will support further system optimization and control to advance the sustainability of microalgal wastewater treatment technologies and biological nutrient recovery.

## 2 Materials and Methods

### 2.1 Full-Scale Treatment System and Long-Term Operation

The Roberts Wastewater Treatment Plant (WWTP; Village of Roberts, WI) has an average influent flow of 410 m^3^·day^–1^ and a design flow of 568 m^3^·day^–1^ for a municipality of nearly 2000 residents.^25^ The Wisconsin Department of Natural Resources decreased the Roberts WWTP’s Wisconsin Pollutant Discharge Elimination System’s (WPDES No. 0028835) 6-month average effluent phosphorus limit from 1 to 0.04 mg-P·L^–1^, effective February 1, 2021, to protect and recover the water quality of the effluent receiving bodies, the East and West Twin Lakes.^26^ The EcoRecover process, which the Village elected to implement for phosphorus removal, was constructed in 2020 and 2021 (Figures 1 and S1). Secondary effluent from sequencing batch reactors (SBRs) is mixed with the microalgal community in a mix tank (average working volume of 98 m^3^ gal) before being sparged with CO_2_ and pumped through five parallel sets of PBRs (77.6 m^3^ total). The PBRs are housed in a greenhouse and, in addition to daylight, received supplemental lighting from above (54 LEDs; California Light Works MegaDrive Centralized Power LED Network) at an intensity of 17 to 50 μE·m^–2^·s^–1^ of photosynthetically active radiation (PAR) measured at the top surface of the PBRs (SI_SCADA.xlsx Supporting Data Set). The separation of HRT and SRT is achieved via submerged, hollow fiber ultrafiltration modules in two parallel membrane tanks with an average transmembrane pressure of 15.4 kPa (Puron ultrafiltration hollow fiber submerged membrane module; Model PHF960; 3.8 m^3^ working volume per train, 18 rows per module, 0.03 μm pores; Koch Separation Solutions, Inc.). A fraction of the permeate is stored in an 11.4 m^3^ reuse tank (average measured HRT of <10 min) for membrane backwashing while the remainder is discharged as effluent. Harvested solids are pumped from the membrane tank and dewatered via centrifugation (Disk Stack Clarifier AC1200–410; Flottweg Separation Technology; Independence, KY). Centrate is mixed in a return tank (6.4 m^3^; average measured HRT of 20 min) with the PBR recycle flow and retentate from the membrane tanks before the combined flow returns to the mix tank.

The mix tank, a nutrient-replete, dark environment, operates as a completely stirred tank reactor and is well mixed through intermittent sparged aeration. Mix tank effluent flows to the PBRs, where the microalgal community is exposed to light, a nutrient-deplete environment, and a single-pass time of 100 min. The median measured hydraulic retention times (HRTs) of the mix tank and PBRs are 3.3 and 4.3 h, respectively. Batch, bench-scale kinetic experiments were conducted for durations that exceeded the single-pass times of the full-scale unit processes to better characterize the kinetics and stoichiometries of nutrient uptake and carbon storage and consumption.

For this study, we focused on monitoring data from November 1, 2022 to February 14, 2023, which represented an extensively sampled period with limited outside influences (e.g., no upstream plant changes, no known chemical perturbation events). Examples of periods with significant outside influence are presented in Sections 3.6 and S6 for transparency but are not the focus of this study.

### 2.2 Bench-Scale Batch Experiments

Bench-scale, batch experiments were conducted to elucidate carbon and nutrient dynamics in the full-scale mix tank and PBRs. Duplicate batch experiments were conducted in cylindrical, bench-scale PBRs constructed from clear cast acrylic with the same diameter as the full-scale system (102 mm inner diameter, 91 cm height, 7 L working volume). The bench-scale PBRs were placed in a greenhouse with the full-scale PBRs to match light and temperature conditions. A third PBR (102 mm inner diameter, 69 cm height, 5 L working volume) was run in parallel, without being sampled, to ensure sufficient solids for the subsequent mix tank bench-scale experiment. Bench-scale mix tank experiments were conducted in duplicate under dark conditions in opaque HDPE plastic containers (4 L working volume) with lids.

Bench-scale experiments were inoculated with biomass and process flows taken directly from the full-scale system immediately before initiation of the batch experiments. The bench-scale PBR experiments were conducted using effluent from the full-scale mix tank. To ensure the biomass in the mix tank batch experiments had adequate stored carbon (at the start of the experiment) to observe organic carbon consumption, the mix tank experiments were conducted using secondary effluent combined with the biomass from the bench-scale PBR that was not sampled (2.2 and 5.8 L, respectively, to match the mixing ratio in the full-scale mix tank).

Reactors were continuously mixed (magnetic stirrer; 300 rpm) and sampled with wide-bore 50 mL serological pipets at 0, 10, 20, 40, 60, 90, 120, 150, 180, and 240 min (PBRs were also sampled at 300 and 360 min). The aqueous fraction of samples was immediately separated from the solids through centrifugation at 4200*g* for 5 min at 4 °C (5804R Eppendorf centrifuge; Enfield, CT) and then filtered through 0.22 μm (MF-Millipore Membrane Filter, 0.22 μm, item no. GSWP02500; MilliporeSigma). The solid pellet and filtered aqueous samples were stored separately at −20 °C prior to lyophilization (solid samples) and analysis (solid and aqueous samples). TSS and volatile suspended solids (VSS) were quantified at 0, 120, and 240 min (as well as at 360 min for the PBRs). The reactor pH was maintained between 6.8 and 7.5 to avoid pH inhibition; adjustments were accomplished with 2 M HCl. The alkalinity of PBR samples—determined via titration of 100 mL samples to pH 4.5 (Mettler Toledo DL55 titrator)—was initially 600 mg·L^–1^ as CaCO_3_ and maintained above 200 mg·L^–1^ as CaCO_3_ through NaHCO_3_ addition to avoid carbon limitation.

### 2.3 Continuous Online Monitoring

For the continuously operating full-scale system, long-term monitoring was achieved through online sensors and analyzers for pH, dissolved oxygen (DO), TSS, PO_4_^3–^, NH_4_^+^, NO_3_^–^, turbidity, temperature, and PAR (Table S1 and Figure S2 in the SI), which interfaced with a SCADA system. Hydraulic parameters, including flow rates and tank volumes, were also collected through online monitoring. Most sensors were online by late November 2020. Following the International Water Association Good Modeling Practice Unified Protocol,^27^ the long-term continuous online monitoring data were reconciled to ensure that systematic errors (e.g., shifts or drifts) in the data set were identified and resolved using the kernel smoothing method of a Python package for functional data analysis (scikit-fda).^28^ In particular, pH, TSS, PO_4_^3–^, NH_4_^+^, and NO_3_^–^ were additionally corrected to match the magnitude of the daily on-site laboratory measurement data (SI_SCADA and SI_AIMS spreadsheets, Supporting Information, (SI).^29^

### 2.4 Aqueous and Suspended Solids Analyses

#### 2.4.1 Long-Term On-Site Sampling and Analysis

Beginning in December 2021, long-term continuous online monitoring was supplemented by analyses of once to twice daily grab and 24 h composite samples from the full-scale system. Aqueous parameters were measured with Hach kits after samples were filtered through 0.45 μm mixed cellulose ester filters. Specifically, aqueous samples were analyzed for orthophosphate and total phosphate (Hach TNT843); alkalinity (TNT870); nitrate (TNT835 or TNT836; dependent on sample concentration range); ammonium (TNT830, TNT831, or TNT832; dependent on sample concentration range); nitrite (TNT839 and TNT840); and total nitrogen (TNT827). The method detection limit (MDL) and minimum reporting level (MRL) for total phosphorus and orthophosphate were estimated according to Ripp 1996,^30^ and were, respectively, found to be 0.005 and 0.005 mg-P·L^–1^ (MDL) and 0.014 and 0.016 mg-P·L^–1^ (MRL; Table S2). Briefly, 9 replicates of the same concentration were analyzed using TNT843, and MDL was defined as the product of the *t*-value for *n*-1 samples (*t* = 2.896) and the sample standard deviation of those replicates. MRL was defined as 3 times the value of the MDL.

#### 2.4.2 Batch, Bench-Scale Analyses

Batch experiment samples (solids and aqueous) were analyzed both on-site and at the University of Illinois Urbana–Champaign (UIUC). Hand-held probes were used to measure pH (Orion 3-Star portable pH meter; Thermo Scientific), temperature, DO (Orion RDO dissolved oxygen probe; Thermo Scientific), and ammonium concentrations (ProDSS multiparameter digital water quality meter; YSI); each sensor was calibrated immediately prior to use in the bench-scale experiments. Solids storage and analyses for TSS and VSS were performed as in Bradley et al.^21,22,31−33^ Briefly, sample TSS was determined by filtration through 0.7 μm glass fiber filters (Whatman GF/F). After filtration, the filters were heated at 105 °C for 1 h and desiccated for 30 min prior to weighing. VSS was determined by combusting samples for 20 min at 550 °C.

Samples for phosphate, nitrate, and nitrite were immediately filtered through 0.22 μm filters and frozen. After storage, aqueous samples were thawed and refiltered prior to analysis via ion chromatography (Dionex ICS-2100 ion chromatograph, Dionex IonPac AS18 column; Section S2 and Figures S3–S5 for calibration curves). The MRL for phosphate was determined to be 0.027 mg-P·L^–1^ and the MRL range was 0.022 to 0.037 mg-P·L^–1^ (Section S2 and Table S3).

### 2.5 Solids Characterization

#### 2.5.1 Elemental Composition

For the elemental analysis of biomass, a solid pellet was collected through centrifugation of a culture sample, immediately frozen, and then lyophilized for 48 h. Phosphorus content was measured through inductively coupled plasma mass spectrometry (ICP-MS; Model NexION 350D, PerkinElmer), and elemental carbon, nitrogen, and hydrogen were measured with a CHN Analyzer (Model CE440, Exeter Analytical) by the UIUC Microanalysis Laboratory. If replicate results had a greater than 5% difference, the sample was reanalyzed, and the results were replaced.

#### 2.5.2 SEM-EDS Biomass Surface Characterization

The surface of lyophilized and ground solids samples were characterized using Scanning Electron Microscopy-Energy Dispersive Spectroscopy (SEM-EDS)^34−36^ in the Microscopy Suite at the Beckman Institute for Advanced Science and Technology at UIUC. Before imaging, the samples were mounted on a stub using carbon tape. The samples were imaged using SEM (Model Quanta FEG 450, FEI company), operating at 15.0 kV and at a working distance of 10 mm. The elemental compositions were measured using an EDAX light-element energy-dispersive spectroscopy system (AMETEK, Inc.) attached to the SEM.

#### 2.5.3 Carbohydrate, Protein, and Lipid Quantification

Solids storage and analyses for protein-to-N ratio and carbohydrate content were performed as in Bradley et al.^21,22,31−33^ Briefly, the protein content was estimated by multiplying the elemental nitrogen content by a conversion factor that represents the ratio of N content to protein. Conversion factors were determined by the analysis of amino acid residuals; amino acid profiling was performed by Bio-Synthesis, Inc. (Lewisville, Texas). Total monomeric carbohydrate content of lyophilized solids was determined after two-step acid hydrolysis of the complete biomass. The hydrolysate was neutralized and filtered, and the monosaccharide concentration was quantified against glucose standards. Solids crude lipid content was quantified as in Gardner-Dale et al.^21,37,38^ Briefly, crude lipids from lyophilized solids were extracted using an adaptation of the Folch method and a 2:1 (v/v) chloroform:methanol solvent mixture. After the extraction, sodium chloride solution was added to bring the final mixture to a 8:4:3 chloroform:methanol:sodium chloride. The mixture was centrifuged, resulting in a biphasic system; the bottom phase containing the crude lipids was transferred to weighing dishes to be measured gravimetrically after the carrier solvent evaporated.

#### 2.5.4 Flow Imaging Microscopy

Mix tank effluent samples (10 mL in a 15 mL conical tube) were collected once daily. Samples were diluted to approximately 1 × 10^6^ particles per milliliter prior to being run on a FlowCam 5000 flow imaging microscope (Yokogawa Fluid Imaging Technologies, Inc.). The resulting collection of detected particles was screened to remove background objects, then used as input data for a deep learning classification model trained on representative libraries of the dominant taxonomic groups observed in the system. Details are given in Section S5.

#### 2.5.5 High-Throughput 18S rRNA Sequencing

1 mL samples of suspended biomass from the mix tank and PBR effluent were collected in triplicate and stored in 5 mL polypropylene transport tubes filled with 3 mL of Zymo DNA/RNA Shield. Samples were kept at −20 °C until they were shipped overnight on ice to the University at Buffalo (UB) SUNY. DNA extraction was performed using the DNeasy Powersoil Pro Kit (Qiagen), and extracts were stored at −20 °C. Polymerase chain reaction (PCR) amplification of the eukaryotic 18S rRNA genes targeted the V8–V9 region (details in Bradley et al.).^39^ Gel electrophoresis was conducted post PCR, and bands of expected size and quality were purified by using the QIAquick gel purification kit (Qiagen). The purified amplicons from each sample were pooled into a DNA library at equimolar proportions (10 ng). Sequencing was performed on the Illumina MiSeq platform with version 3 chemistry (300-cycle paired-end reads) at the UB Genomics and Bioinformatics Core. Raw sequencing data are available on NCBI under BioProject accession number PRJNA1045645. The sequencing read processing (i.e., quality filtering and trimming, taxonomic assignment) and statistical analyses (including alpha and beta diversity) were conducted following the established protocol (MiSeq SOP) provided by mothur v1.48.0.^40^

## 3 Results and Discussion

### 3.1 Phosphorus Removal Over Time

**Figure 2:**
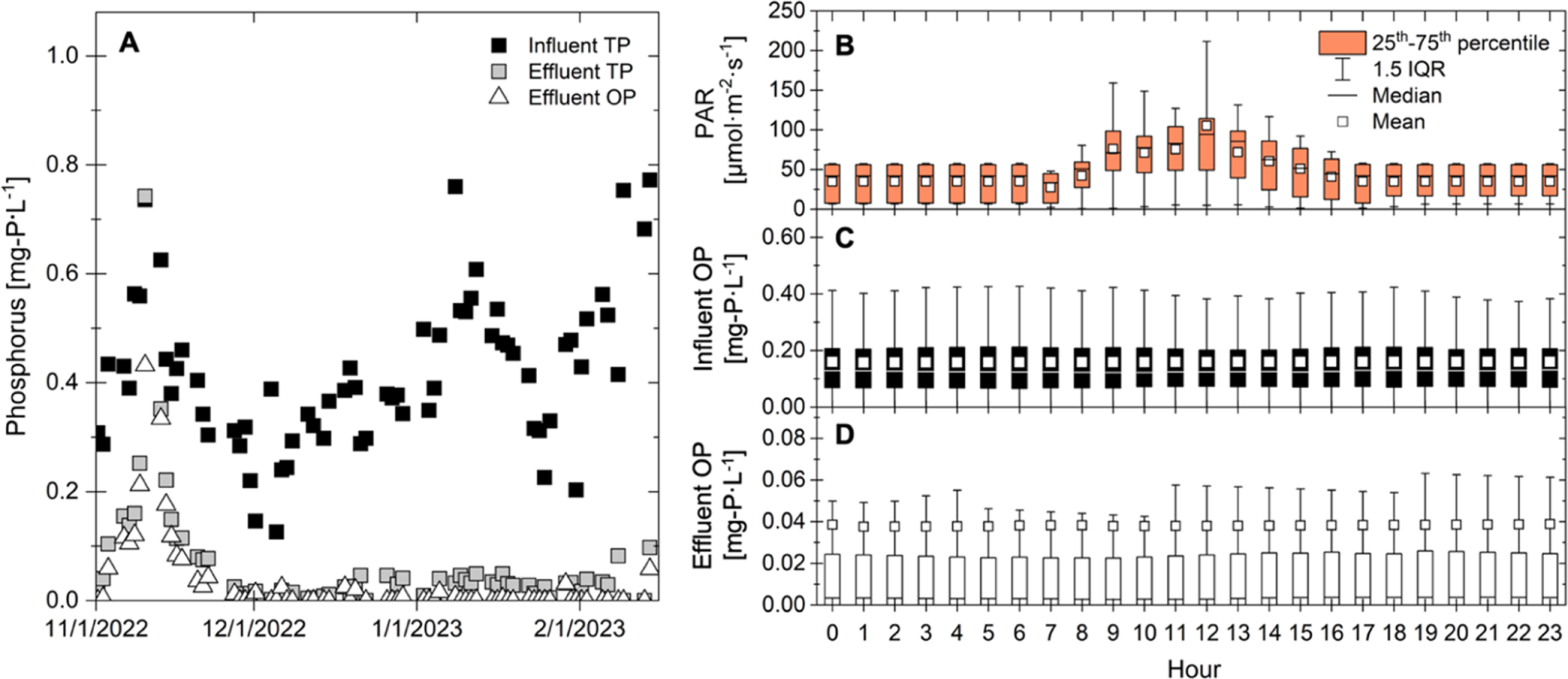
(A) Influent and permeate total phosphorus (TP) concentrations and permeate orthophosphate (PO_4_^3–^) concentrations for the EcoRecover process in the winter from November 1, 2022 to February 14, 2023. 24 h composite samples were collected and immediately analyzed by an on-site laboratory technician (EH). (B–D) Diel variability of hourly (B) photosynthetically active radiation (PAR), (C) influent orthophosphate (OP) concentration, and (D) effluent OP concentration across the full focus period (November 1, 2022 through February 14, 2023).

The first full-scale installation of the EcoRecover process at Roberts, Wisconsin demonstrated phosphorus recovery from secondary effluent via microalgal biomass cultivation 24 h per day and across seasons. The focus period (November 1, 2022 through February 14, 2023; 106 days) began with a 2-week upset and recovery period, followed by 92 days (November 15, 2022 through February 14, 2023) of superior performance in which the system continuously achieved effluent (permeate) orthophosphate concentrations below 0.04 mg-P·L^–1^ (Figure 2A). Across the full focus period, the effluent total phosphorus concentration of 24 h composite samples averaged 0.06 ± 0.11 mg-P·L^–1^ (0.03 ± 0.08 mg-P·L^–1^ orthophosphate; average ± standard deviation); within the 92-day period of excellent performance, the effluent total phosphorus concentration averaged 0.03 ± 0.03 mg-P·L^–1^ (0.01 ± 0.02 mg-P·L^–1^ orthophosphate; Figure S6). The influent ammonium concentration shifted significantly over the focus period as the upstream SBRs lost nitrification in December 2022 and total nitrogen increased in January 2023 (Figures S7 and S8). As a result, effluent ammonia concentrations were highly variable (Figure S7). Within the EcoRecover process, some nitrification was observed from November 1 to December 8, 2022, but nitrification was negligible from December 8, 2022 to February 14, 2023 (Figures S8 and S9). The EcoRecover process was implemented specifically to achieve phosphorus removal, but future work could target sustained nitrification or integrate the system with complementary processes for nitrogen conversion or removal (e.g., denitrification filters).

Photosynthetically active radiation (PAR)—a measure of the photon flux density at wavelengths that are available for photosynthesis—varied over 24 h cycles from a mean of 27.5 to 105 μE·m^–2^·s^–1^, with the highest intensities observed at noon (Figure 2B). Regardless of diel irradiance patterns, influent orthophosphate was relatively stable across the day with hourly mean concentrations that ranged from 0.157 to 0.161 mg-P·L^–1^ (Figure 2C), and effluent orthophosphate was consistent with average values of 0.038 to 0.039 mg-P·L^–1^ (Figure 2D). Thus, phosphorus removal was stable across 24 h cycles, including across daily periods (15+ h) of low irradiance.

### 3.2 Role of Storage Carbohydrates in Phosphorus Uptake

**Figure 3:**
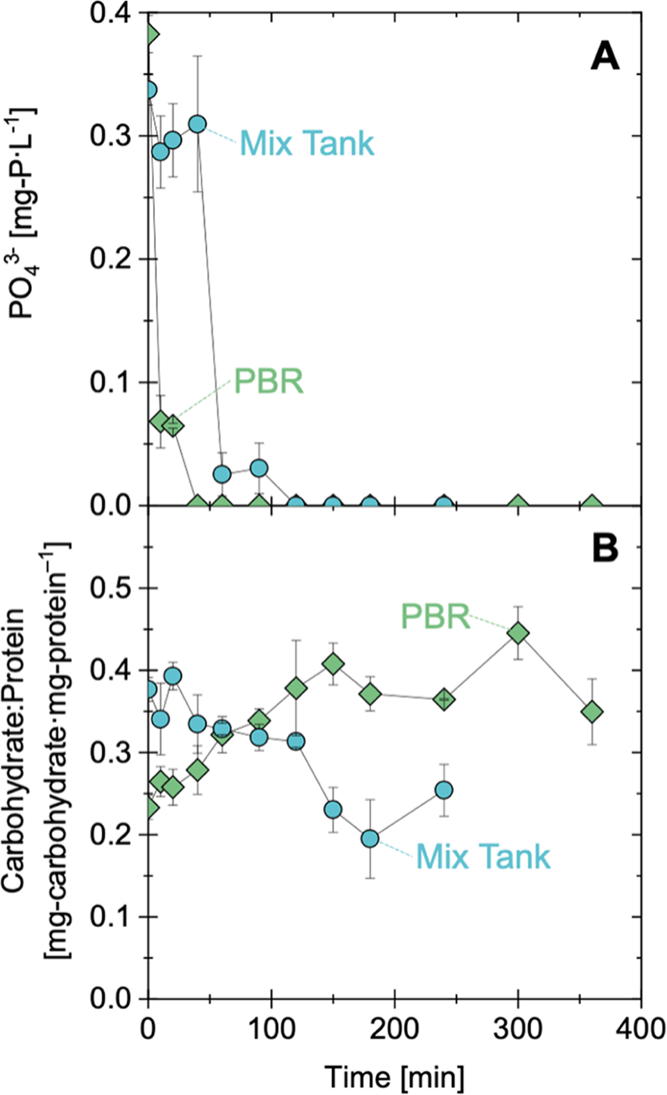
(A) Orthophosphate and (B) carbohydrate:protein ratios in bench-scale batch experiments to mimic conditions in the mix tank (blue circles) and PBRs (green diamonds). Batch experiments were performed in duplicate. The duration of the experiments was at least twice the HRT of the full-scale unit processes. Mix tank experiments were carried out with bench-scale PBR culture combined with a full-scale secondary effluent. PBR experiments were carried out with full-scale mix tank culture. Symbols represent averages, with error bars extending to individual replicate (i.e., minimum and maximum) values.

Past work has demonstrated that carbohydrate storage and consumption (also referred to as carbohydrate mobilization) is important for the uptake of nutrients across diel light/dark cycles in suspended algal cultures and communities.^21,22^ Specifically, exposure to lit, nutrient-limited conditions can induce the storage of biopolymers, which can then be leveraged under dark conditions to support nutrient uptake and continued metabolic activity. To determine whether the microbial community had the capacity to balance metabolic activity under the more rapid light/dark cycling observed across EcoRecover unit operations, batch experiments were conducted on-site under mix tank (dark) and PBR (illuminated) conditions in May 2022 (prior to examining the extant, full-scale carbon and nutrient dynamics). In the simulated PBR (performed with EcoRecover mix tank effluent), the initial orthophosphate of 0.383 ± 0.015 mg-P·L^–1^ was removed within 40 min (Figure 3A), and the initial ammonium concentration of 36 ± 4 mg-N·L^–1^ was reduced to 5.24 ± 0.13 mg-N·L^–1^ over 360 min (Figure S20). As expected, phosphorus limitation and the availability of light in the simulated PBR resulted in carbohydrate storage and an increase in the carbohydrate/protein ratio of solids (Figure 3B). Consistent with this observation, the solids C/N and C/P mass ratios increased (Figures S22 and S23, respectively) and PBR solids concentrations increased (from a VSS of 470 ± 40 to 700 ± 20 mg·L^–1^ over 360 min; Figure S24). This photosynthetic fixation of inorganic carbon also resulted in oxygen production, with a significant increase in DO over time (Figure S25).

In the simulated mix tank (performed with simulated PBR culture mixed with EcoRecover influent), the initial orthophosphate of 0.337 ± 0.012 mg-P·L^–1^ was removed within 120 min (Figure 3A), and the initial ammonium concentration of 14 ± 2 mg-N·L^–1^ was reduced to 8.90 ± 0.01 mg-N·L^–1^ over the course of 240 min (Figure S20). The dark conditions in the simulated mix tank resulted in carbohydrate consumption, which was observed as a decrease in solid carbohydrate/protein (Figure 3B), C/N (Figure S22), and C/P (Figure S23) mass ratios over time accompanied by the consumption of DO (Figure S25). Although storage carbohydrates were consumed, a net change in VSS was not observed in the mix tank (two-sample *t* test, two-tailed, *p* = 0.876; Figure S24).

**Figure 4:**
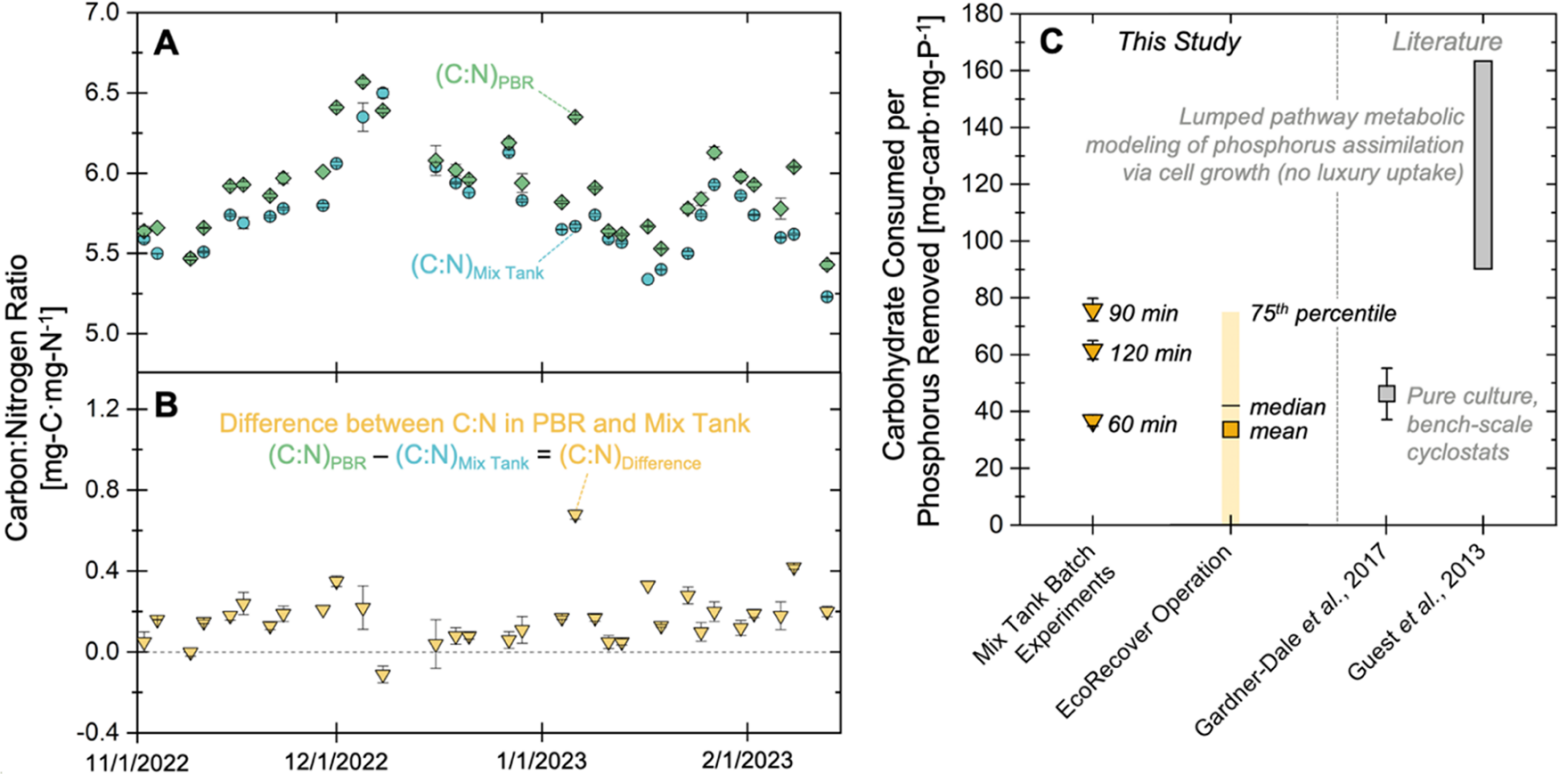
(A) Solids C/N ratio (by mass) in the full-scale mix tank (blue circles) and PBR (green diamonds) effluent from November 1, 2022 to February 14, 2023. Error bars represent relative error from analytical duplicates. (B) Difference between the PBR and mix tank solids C/N ratios, with positive values supporting the hypothesis that the microbial community was storing carbohydrates in the PBRs and mobilizing (i.e., consuming) stored carbohydrates in the mix tank. Error bars in panel (B) represent propagated relative error (eq S1) from analytical duplicates in panel (A). (C) Mass of carbohydrates consumed per mass of phosphorus removed in this study and in the literature. Ratios for mix tank batch experiments were calculated at 3 time points (60, 90, and 120 min), and full-scale EcoRecover operational results were calculated based on 25 time points for which carbohydrate and total phosphorus removal data were available. Additional details regarding calculations for (C) can be found in Section S7 (Bench-scale batch experiments) and Section S8 (Full-scale biomass composition trends).

To determine whether these mechanisms occur across units during the continuous operation of the EcoRecover process, biomass samples were collected from the mix tank effluent and PBR effluent across the full focus period. In the full-scale system, higher biomass C/N ratios (and carbohydrate/protein ratios) were consistently observed in the PBR effluent than the mix tank effluent during periods of good performance (Figures 4A,B and S28). The observation of higher C/N ratios in biomass leaving the PBRs relative to the biomass leaving the mix tank further underscores the importance of stored carbohydrates in nutrient recovery and the balancing of cell growth across unit operations. To benchmark the observed carbohydrate consumption across batch and continuous operations, the mobilized carbohydrates were normalized to the quantity of phosphorus recovered from the system. The microbial communities consumed between 37 and 76 mg-carbohydrate·mg-P^1–^ in the mix tank batch experiment (after 60 to 120 min) and an average of 33 mg-carbohydrate·mg-P^1–^ (median of 42 mg-carbohydrate·mg-P^1–^; *n* = 25) during continuous operation across the full focus period (Figure 4C). These ratios are similar to past values reported in the literature for bench-scale phosphorus-limited experiments with *Scenedesmus obliquus* and *Chlamydomonas reinhardtii* (46 ± 9 mg-carbohydrate·mg-P^1–^),^21^ and less than the theoretical ratio derived from lumped pathway metabolic modeling (90 to 163 mg-carbohydrate·mg-P^1–^; Figure 4C).^23^ One potential explanation for lower carbohydrate consumption (relative to theoretical values) is the storage of polyphosphate, which would coincide with phosphorus uptake but would not have the same energy, reducing power, and carbon requirements as cell growth processes (including the synthesis of functional biomass precursors^23^). Altogether, results from the bench-scale experiments and full-scale monitoring confirmed that (i) phosphorus removal occurs in dark, nutrient-rich conditions (i.e., the mix tank) and is facilitated by the consumption of stored carbohydrates and oxygen, (ii) photosynthesis in the PBRs supports the uptake of residual phosphorus, the accumulation of carbohydrates in cell biomass, and the production of oxygen, and (iii) phosphorus uptake and growth may be (at least) partially decoupled (e.g., via luxury uptake of phosphorus).

### 3.3 Luxury Uptake of Phosphorus

To explore alternative mechanisms for phosphorus recovery, the elemental composition of harvested biomass was characterized. The phosphorus content in algal biomass has been shown to vary across species^41,42^ and also within species as a function of their physiological state.^43−47^ The phosphorus content in microalgal cells is often around 1% of dry weight under limited phosphorus availability^48^ but may be as high as 3–10% dry weight when the microalgae achieve luxury uptake of phosphorus.^14,19,49,50^ The average phosphorus content of harvested biomass across the full focus period was 3.2 ± 0.7% (5/50/95th percentiles of 2.2/3.6/4.0%; *n* = 32; Figure S13), further suggesting partial phosphorus recovery via luxury uptake, surface adsorption, or precipitation of phosphorus.

**Figure 5:**
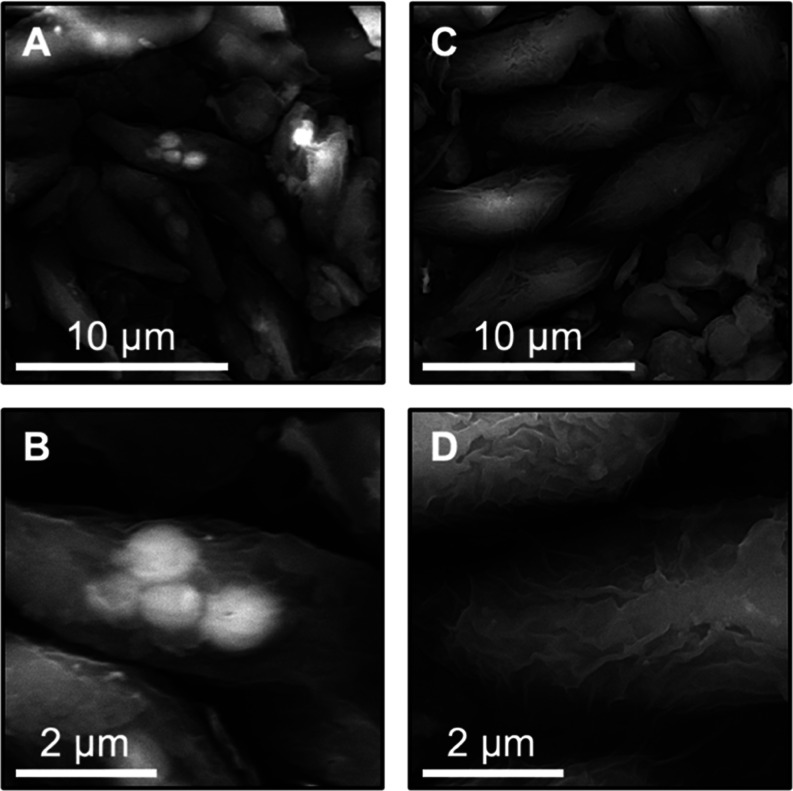
(A–D) SEM-EDS images of lyophilized and ground EcoRecover solids from (A, B) the highest observed phosphorus content and (C, D) the lowest observed phosphorus content based on elemental analysis (Table S5). Bright particles in panels (A, B) represent highly localized, elevated concentrations of phosphorus and metals that appear as granules within cells.

SEM-EDS was performed on a range of samples from the broader monitoring period (April 5, 2022 to February 13, 2023; Section S4.2 and Table S5), including samples with the highest and lowest phosphorus content, from periods with and without coagulant use, and start and end points of batch experiments. Of selected samples for SEM-EDS imaging, the February 13, 2023, sample had the greatest overall phosphorus content (5.1%). Phosphorus-rich granules were observed in biomass samples and appeared to be within microalgal cells (Figure 5A,B), supporting the hypothesis that elevated phosphorus could be due to polyphosphate accumulation within cells. Additional evidence of polyphosphate accumulation was seen in EDS measurements, which showed a higher phosphorus content in granules than in biomass (i.e., cell components away from granules) and indicated a positive relationship between phosphorus and cation content (Figure S16); a positive correlation between phosphorus and cation content is consistent with past studies analyzing polyphosphate-accumulating organisms.^51,52^ Finally, the dominant taxa at this date was *Scenedesmus spp.* (discussed below), which is capable of polyphosphate accumulation.^45^ In periods of low biomass phosphorus content (e.g., 1.3% on August 16, 2022), no granules were observed (Figure 5C,D). Beyond polyphosphate accumulation, high pH at the surface of microalgal cells (due to inorganic carbon fixation) may have facilitated inorganic phosphorus precipitation on the cell surfaces or within extracellular polymeric substance (EPS) even at low total phosphorus concentrations.^53−55^ This mechanism of phosphorus recovery, however, was not as readily apparent.

### 3.4 Nitrogen to Phosphorus Ratios

The nitrogen content of solids was less variable than that of phosphorus. Across the focus period, the average biomass yield was 12.8 ± 0.7 kg TSS·(kg-N)^−1^, corresponding to a solids nitrogen content of 7.8 ± 0.4% (average ± standard deviation; 5/50/95th percentiles of 7.10/7.82/8.49%). Similar to phosphorus, nitrogen content of microalgae can vary, with reported nitrogen content ranging from 5.87 to 11.16% of total solids.^56,57^

N/P mass ratios across the focus period were 2.5 ± 0.6 (Figure S17) but were notably higher prior to January 8, 2023 (3.3 ± 0.3, with phosphorus content of 2.4 ± 0.2%) than after (2.1 ± 0.2; with phosphorus content of 3.7 ± 0.4%). The nitrogen content remained relatively stable at 7.8 ± 0.5 to 7.9 ± 0.4% for the periods before and after January 8, 2023, respectively. Solids N/P ratio may vary with growth rate (linked to SRT) and influent N/P ratio through interspecific stoichiometric plasticity (especially under nutrient-limitation) or microbial community composition shifts in favor of species that have a competitive advantage in a given set of environmental conditions.^21,47,58^ The relationship between influent N/P and solids N/P was not significant (linear *R*^2^ < 0.001, *n* = 12). However, solids N/P had a negative linear correlation with SRT (*R*^2^ = 0.63, Figure S18) during the period of superior performance (November 15, 2022 to February 14, 2023), varying from 2.1 to 4.2 days. In addition to SRT, the biomass N/P ratio may have also been influenced by the fate of recovered phosphorus, which varied between assimilation within the cells and highly localized precipitation on the cell surfaces or within the EPS during upstream coagulant use.

### 3.5 Community Structure Dynamics

**Figure 6:**
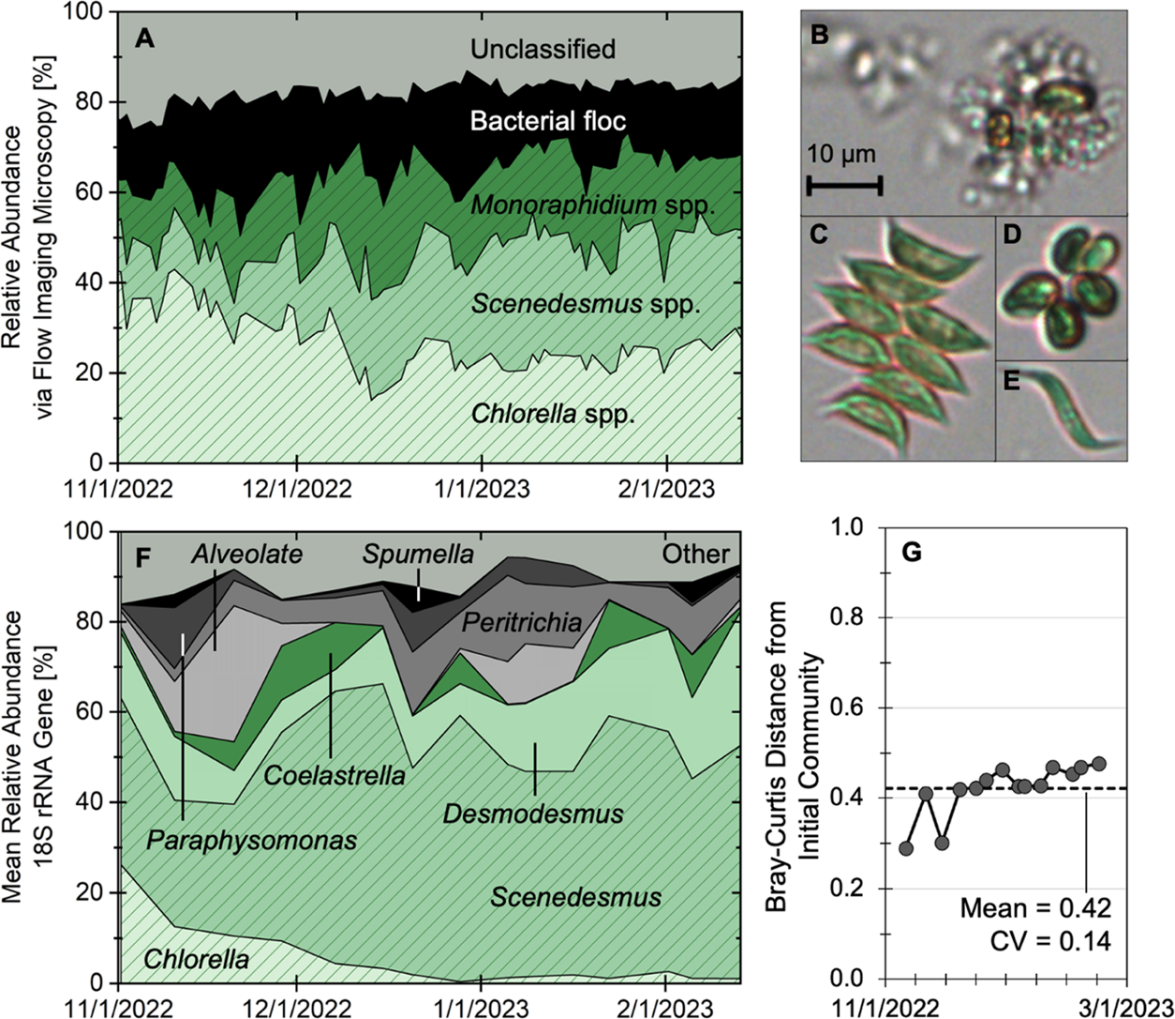
(**A**) Mixed microbial community taxa distribution for the EcoRecover process from November 1, 2022 to February 14, 2023. (B–E) FlowCam images of the four dominant categories identified via flow imaging microscopy during this period: (B) bacterial floc; (C) *Scenedesmus spp.*; (D) *Chlorella spp.*; and (E) *Monoraphidium spp.* (F) Mean relative abundance (MRA) of the top eukaryotic genera from 18S rRNA sequencing. (G) Bray–Curtis distances of each sampling point from the initial eukaryotic community on November 2, 2022.

Across the entire focus period, the algal community was relatively stable. Flow imaging microscopy identified *Chlorella spp.*, *Scenedesmus spp.*, and *Monoraphidium spp*. as the dominant constituents of the microalgal community (Figure 6A–E). High-throughput sequencing of 18S rRNA genes confirmed the eukaryotic community was dominated by green microalgae, including *Scenedesmus* (28–63%), *Desmodesmus* (5–31%), and *Chlorella* (0.5–26.5%; Figure 6F). The eukaryotic community remained stable during this period (Figure 6G), as indicated by a consistent Bray–Curtis dissimilarity among samples across the intensive sampling period from the starting community on November 2, 2022 (mean = 0.42, std. dev. = 0.06, and coeff. of variation = 0.14; Figure 6G). Additionally, when compared to a variable performance period (February 15, 2023 to April 28, 2023) immediately following the focus period, eukaryotic communities in these two periods showed significantly different clusters (*p* < 0.01, AMOVA)^59^ as illustrated by ellipses encompassing 95% of cluster assigned data points (Figure S10). Full, longer-term sequencing results and more in-depth community structure analyses are the focus of a separate study.^60^

### 3.6 General Process Stability and Upset Events

Key indicators of stable performance included near-neutral pH throughout the system, sufficient residual alkalinity in the EcoRecover effluent (>100 mg·L^–1^ as CaCO_3_), and a steady daily rhythm in the DO concentration in the PBR effluent (Figure S11). In particular, the cycling of the DO concentration in the PBR effluent was a readily available indicator for operational staff, given that the DO concentration followed the same pattern as diel lighting intensity when the microbial community is photosynthetically active (Figures 2B and S12), often reaching 10–20 mg·L^–1^ of DO during peak daylight hours. Although the mix tank was intermittently sparged with air to achieve mixing, the combination of sparging, influent DO (from SBR effluent, internal recycle from the PBR effluent, return activated algae [RAA] from the membrane tank, and centrate; Figure S2), and oxygen consumption (which accompanies stored carbohydrate consumption) resulted in relatively stable DO in the mix tank across individual day-night cycles (Figure S11).

Periods of process upset or variable performance were defined as periods of prolonged or intermittent exceedance of the 0.04 mg-P·L^–1^ effluent target, respectively. Although not the focus of this study, the system was susceptible to process upsets driven by upstream changes to the wastewater treatment process (e.g., reduction in SBR settling time that resulted in TSS concentrations >100 mg-TSS·L^–1^ entering the EcoRecover process), high influent concentrations of a disinfectant (quaternary ammonium), and biological drivers (including grazers^60^). Periods of process upset or variable performance were often characterized by variable or basic pH, loss of DO diel rhythm (e.g., as seen in November 2022, Figure S11), and at times included insufficient alkalinity and solid composition changes. Additional details of process upsets are provided in Section S6 of the SI, and two specific examples are discussed in detail by Alam and colleagues for a separate study period (November 2021 through August 2022).^60^

### 3.7 Path Forward for Intensive, Suspended Growth Microalgal Wastewater Treatment

Wastewater resource recovery facilities that support urban populations are often landlocked and need to intensify their processes to meet more stringent effluent permits or increase their treatment capacity.^61^ Adoption of high productivity, small footprint (intensive) microalgal technologies creates an opportunity to sustainably convert waste nutrients to marketable products and meet rigorous effluent nutrient criteria. While EBPR may remove phosphorus to effluent concentrations approaching 0.1 mg-P·L^–1^, the EcoRecover process has demonstrated long-term recovery of phosphorus to achieve effluent total phosphorus concentrations below 0.03 mg-P·L^–1^, even in the winter months in Wisconsin (latitude of 45° N). An additional advantage of algal-based systems is the potential for organic phosphorus (and organic nitrogen) recovery,^11^ which remains a critical challenge for conventional bacterial and precipitation-based nutrient removal technologies.^62^ Future studies may specifically focus on organic nutrient recovery, as well as the sustainability implications (e.g., reduced chemical dosages and CO_2_ sequestration, increased process energy consumption)^63^ of replacing alternative tertiary treatment processes such as chemical phosphorus polishing. Through the integration of algal process models (e.g.,^23^) with techno-economic analysis and life cycle assessment, future work may characterize the financial, environmental (including effluent quality, greenhouse gas emissions, etc.), and energy implications of process design decisions and deployment scenarios to guide future research and investment.

In the algal cultivation space, technologies are often compared based on their areal productivities. In this study, the characterized EcoRecover process was intentionally designed to be phosphorus-limited to meet stringent permit requirements. As a result, the system was not designed to maximize biomass productivity and instead prioritized reliable effluent quality (with biomass production and sale serving as a secondary benefit). Nonetheless, across the focus period (November 1, 2022 to February 14, 2023), the EcoRecover’s areal productivity was 15 ± 4 g·m^–2^·day^–1^ in winter months (external temperatures from −27 to 24 °C, daily average of −7 °C) at a high altitude (45° N). In other monitored periods subject to upstream upsets or other external pressures (e.g., chemical shortages), areal productivities on the order of 45 g·m^–2^·d^–1^ (average from July 26 to September 6, 2022; individual time point estimates ranged from 36 to 60 g·m^–2^·d^–1^) were also observed.

Ultimately, this work represents the first full-scale characterization of the EcoRecover process for algae cultivation and tertiary nutrient recovery. Future work will continue to build off this understanding to advance our ability to optimize the design of this system, mechanistically and dynamically model its performance, and develop tailored solutions for utilities seeking to simultaneously advance goals for improved effluent quality and engagement with the circular bioeconomy.

## Supporting Information Available

Images of the EcoRecover system at Roberts, WI, USA; detailed descriptions of online monitoring equipment; expanded continuous monitoring results for system performance; detailed description of ion chromatography analyses; descriptions of periods of system performance upset; solids characterization through SEM-EDS; batch experiment aqueous and biomass analyses (PDF)
Cleaned long-term AIMS monitoring data (XLSX)
Cleaned long-term SCADA monitoring data (XLSX)
