# Supplementary material for: Intensive Microalgal Cultivation and Tertiary Phosphorus Recovery from Wastewaters via the EcoRecover Process
Source: Environ Sci Technol. 2024 Apr 30;58(20):8803–14. doi: 10.1021/acs.est.3c10264 (PMC11112746; doi:10.1021/acs.est.3c10264)
Supplement: Supplementary file 1 — es3c10264_si_001.pdf [file es3c10264_si_001.pdf]

Supporting Information for

Intensive Microalgal Cultivation and Tertiary Phosphorus Recovery from Wastewaters via the EcoRecover Process

Hannah R. Molitor,[1] Ga-Yeong Kim,[1] Elaine Hartnett,[2] Benjamin Gincley,[3] Md Mahbubul Alam,[4] Jianan Feng,[1] Nickolas M. Avila,[1] Autumn Fisher,[2] Mahdi Hodaei,[4] Yalin Li,[5,6] Kevin McGraw,[2] Roland D. Cusick,[1] Ian M. Bradley,[4,7] Ameet J. Pinto,[3] Jeremy S. Guest[1,5,*]

[1] Department of Civil & Environmental Engineering, Newmark Civil Engineering Laboratory, University of Illinois Urbana-Champaign, Urbana, IL, 61801, USA.

[2] Clearas Water Recovery, Inc., Missoula, MT 59808, USA.

[3] School of Civil and Environmental Engineering, Georgia Institute of Technology, Atlanta, GA 30332, USA.

[4] Department of Civil, Structural and Environmental Engineering, University at Buffalo, The State University of New York, Buffalo, NY 14260, USA.

[5] Institute for Sustainability, Energy, and Environment, University of Illinois Urbana-Champaign, Urbana, IL 61801, USA.

[6] Department of Civil and Environmental Engineering, Rutgers, The State University of New Jersey, Piscataway, NJ 08854, USA.

[7] Research and Education in Energy, Environmental and Water (RENEW) Institute, University at Buffalo, The State University of New York, Buffalo, NY 14260, USA.

*Corresponding author: jsguest@illinois.edu, +1 (217) 244-9247

28 Figures and 5 Tables

Table of Contents

S1. Full-scale treatment system and long-term continuous monitoring S4
S2. Calibration and determination of MRL and MDL S6
S3. Characterization of long-term continuous performance S9
S4. Full-scale biomass composition trends S13
S5. Flow imaging microscopy S18
S6. Susceptibility to process upsets S19
S7. Bench-scale batch experiments S20
S8. Full-scale biomass composition trends S24

List of Tables

Table S1. Measurement units, instrument model and manufacturer information, frequency of data collection, and sensor location for measurements made across the EcoRecover system with on-line sensors and analyzers. Affiliated with Figure S1.

Table S2. Determination of the MDL for orthophosphate and total phosphorus via Hach TNT843

Table S3. Results from PO_4-P IC MDL work: std dev of lowest 3 standards and 0.5 mg/L PO4-P, slope and y-intercept of calibration curve, R^2 value, etc.

Table S4. EcoRecover mix tank and PBR biomass phosphorus content metrics.

Table S5. Criteria for sample selection for SEM-EDS analysis and key system operating conditions and performance during the sampling date.

List of Figures

Figure S1. Images of the CLEARAS EcoRecover system at the Village of Roberts Wastewater Treatment Plant.

Figure S2. Schematic of sensor and analyzer locations throughout the EcoRecover system at the Village of Roberts Wastewater Treatment Plant. Affiliated with Table S1.

Figure S3. Calibration curve for orthophosphate as measured on a Dionex ICS2100 anion ion chromatograph with a matrix background.

Figure S4. Calibration curve for NO_3^--N and peak area as measured on a Dionex ICS2100 anion ion chromatograph.

Figure S5. Calibration curve for NO_2^--N and peak area as measured on a Dionex ICS2100 anion ion chromatograph.

Figure S6. Orthophosphate and total phosphorus concentrations in the influent and effluent of the EcoRecover system for the period between November 1, 2022, to February 14, 2023.

Figure S7. NH_4^+-N concentration in the EcoRecover system influent (secondary effluent) and permeate for the period of November 1, 2022 to February 14, 2023.

Figure S8. NO_3^--N concentration in the EcoRecover system influent (secondary effluent) and permeate for the period of November 1, 2022 to February 14, 2023.

Figure S9. Difference in oxidized nitrogen species (NO_3^--N and NO_2^--N) concentrations in EcoRecover effluent and influent 24-h composite samples for the period of November 1, 2022 to February 14, 2023.

Figure S10. Non-metric multi-dimensional scaling plot showing the spatial distances among eukaryotic communities throughout the focus period.

Figure S11. Dissolved oxygen concentrations in the mix tank and photobioreactor effluent for the period of November 1, 2022 to February 14, 2023.

Figure S12. Photosynthetically active radiation (PAR) at four locations on the photobioreactors for the period of November 1, 2022 to February 14, 2023.

Figure S13. Biomass phosphorus content in the EcoRecover mix tank and PBR effluent mixed microalgal culture from February 21, 2022 to May 12, 2023.

Figure S14. SEM-EDS images of lyophilized and ground EcoRecover PBR biomass during a period of coagulant use.

Figure S15. SEM-EDS images of lyophilized and ground EcoRecover PBR biomass during 4-week period without coagulant use.

Figure S16. Phosphorus and metals (sum of Na, Mg, Al, K, Ca) weight percentages of analyzed granules and biomass based on SEM-EDS.

Figure S17. Biomass N:P mass ratio for the period between November 1, 2022 to February 14, 2023.

Figure S18. The EcoRecover biomass N:P mass ratio had a negative linear correlation with the system SRT during a 3-month period of excellent performance.

Figure S19. Comparison of EcoRecover culture during and after a process upset.

Figure S20. Average NH_4^+-N removal over time in batch, bench-scale mix tank and PBR duplicate experiments

Figure S21. Average NO_3^--N and NO_2^--N concentrations over time in batch, bench-scale mix tank and PBR duplicate experiments.

Figure S22. Carbon:nitrogen (C:N) mass ratio for bench-scale experiments that replicated the conditions of the mix tank and PBRs.

Figure S23. Carbon:phosphorus (C:P) mass ratio for bench-scale experiments that replicated the conditions of the mix tank and PBRs.

Figure S24. Volatile suspended solids (VSS) concentration for bench-scale experiments that replicated the conditions of the mix tank and PBRs.

Figure S25. Dissolved oxygen concentrations over time for bench-scale experiments that replicated the conditions of the mix tank and PBRs.

Figure S26. Crude lipid to protein mass ratio for bench-scale experiments that replicated the conditions of the mix tank and PBRs.

Figure S27. Nitrogen:phosphorus (N:P) mass ratio for bench-scale experiments that replicated the conditions of the mix tank and PBRs.

Figure S28. Biomass carbohydrate:protein ratio in the full-scale Mix tank and PBR effluent, and the difference between the PBR and mix tank biomass carbohydrate:protein ratio during November 1, 2022 to February 14, 2023.

List of Equations

Equation S1. Propagation of error for subtraction of Mix tank and PBR duplicate analyses.

S1. Full-scale treatment system and long-term continuous monitoring

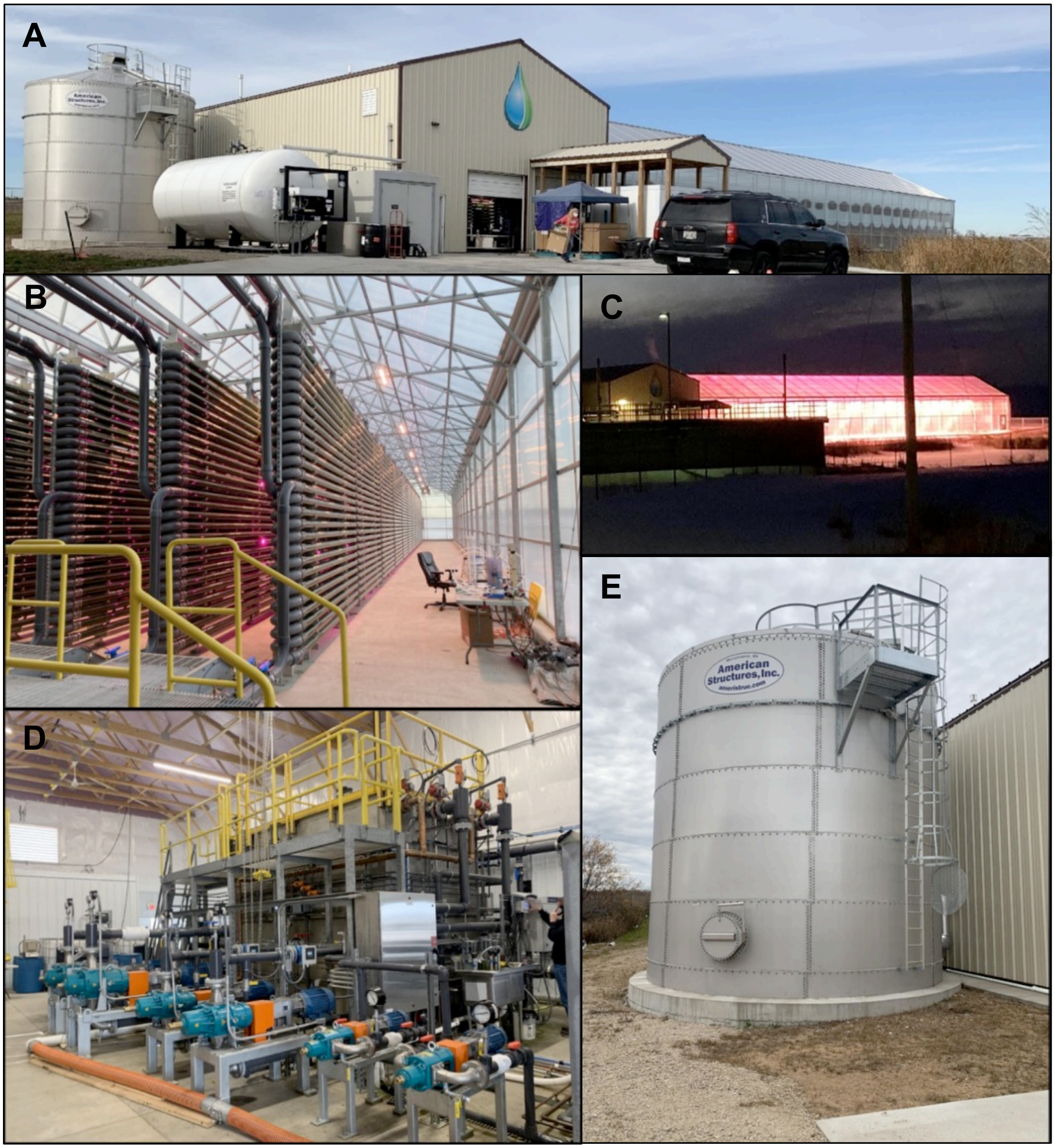

Figure S1. Photographs of the Village of Roberts EcoRecover system, including (A) the exterior of the plant showing the mix tank, CO_2 storage tank, equipment building, and greenhouse (left to right), (B) the PBR fences in the interior of the greenhouse, (C) the greenhouse lit by supplemental lighting at night, (D) the system block pumps and housing for the membrane tanks and return tank, and (E) the mix tank located externally to the equipment building.

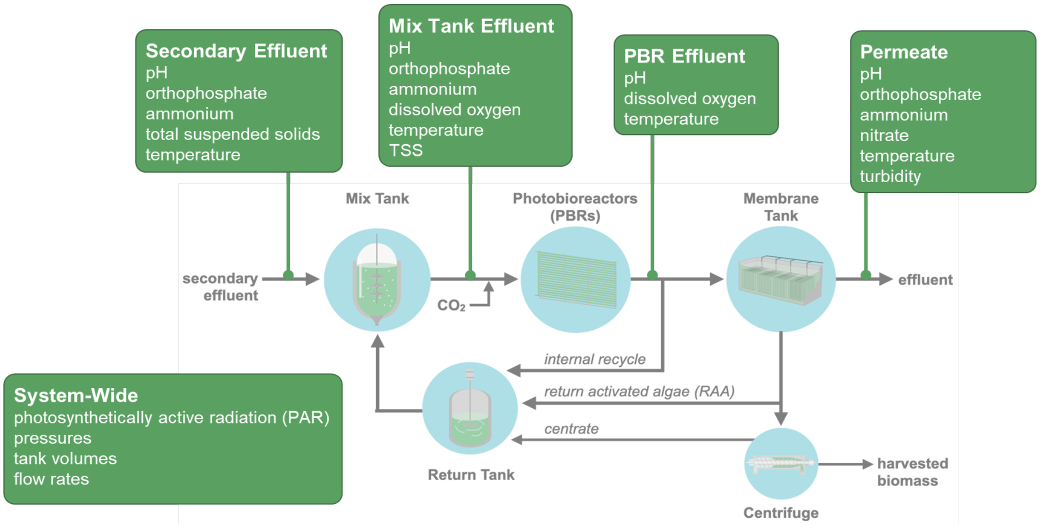

Figure S2. Schematic of sensor and analyzer locations throughout the full-scale EcoRecover system.

Table S1. Measurement units, instrument model and manufacturer information, frequency of data collection, and sensor location for measurements made across the full-scale EcoRecover system with on-line sensors and analyzers.

Measurement [units]	Sensor/Analyzer	Frequency of Data	Sensor Location	Calibration**,***
Ammonium [$mg{\cdot}L^{-1}$]	AmmoLyt Plus 700 IQ, #107072YK, YSI Inc.	5 min	Secondary Effluent, Mix tank Effluent	Matrix adjusted calibration, once per month or as needed
Ammonium [$mg{\cdot}L^{-1}$] and Nitrate [$mg{\cdot}L^{-1}$], combined sensor	VARiON Plus 700 IQ Probe, #107680YK, YSI Inc.	5 min	Permeate	Matrix adjusted calibration, once per month or as needed
Dissolved oxygen [$mg{\cdot}L^{-1}$]	IQ SensorNet FDO 700 IQ Optical Dissolved Oxygen Probe, #201650Y, YSI Inc.	5 min	Mix tank Effluent, PBR Effluent	N/A
Orthophosphate	Alyza IQ PO4, #852211Y (single channel) and #852212Y (two channels), YSI Inc.	5 min	Secondary Effluent, Mix tank Effluent, Permeate	Automatic daily internal calibration
PAR [$\mu mol{\cdot}m^{-2}{\cdot}s^{-1}$]	Model SQ-214 PAR sensors, Apogee Inc.	5 min	PBR Greenhouse (PBR fences 2 and 4 each have one high and one low)	N/A
pH and Temperature [°F]	pH IQ SensorNet Probe, #109170Y, with pH Electrode, #109115Y, YSI Inc.	5 min	Secondary Effluent, Mix tank Effluent, PBR Effluent, Permeate	Calibration using buffer solutions, once per month or as needed
TSS [$mg{\cdot}L^{-1}$]	ViSolid 700 IQ SensorNet TSS probe, #600012Y, YSI Inc.	5 min	Mix tank Effluent	N/A
Turbidity [NTU]	TU5300 sc Turbidimeter, Hach Co.	5 min	Permeate	N/A
Flowrate [GPM]	Proline Promag electromagnetic flowmeters, Endress & Hauser	5 min	Secondary Effluent, Mix tank Effluent, PBR Effluent (3 fences), Internal Recycle, Combined Membrane Influent, Centrifuge Influent, Tertiary Effluent (from 2 membrane trains)	N/A
Tank Volume [gal]	FLT93B FlexSwitch, Fluid Components International LLC	5 min	Mix tank, Membrane Tank (2 trains), PBRs,* Return Tank, Reuse Tank	N/A
Tank Level	Waterpilot FMX21, Endress & Hauser	5 min	Membrane Tank (2 trains)	N/A

*The PBR total volume was constant at 20,470 gal (full capacity) and was recorded at an average interval of 9 h.
**Sensor and analyzer calibration times varied depending on the manufacturer's recommendations and the sensor performance.
***Probes were cleaned three times per week by rinsing with tap water and gently wiping with a delicate task wipe (Kimwipes).

S2. Calibration and determination of MRL and MDL

Table S2. Determination of the MDL and MRL for orthophosphate and total phosphorus via Hach TNT843 for nine replicates each of 0.01 mg-P·L^{-1} as PO_4^{3-}. The method reporting limit (MRL) was 0.014 and 0.016 mg-P·L^{-1} as PO_4^{3-}, total phosphorus and orthophosphate, respectively. The MDL was calculated as the standard deviation of nine replicates (n = 9) multiplied by the t-value for n - 1 samples (t-value = 2.896). The MRL was 3-times the MDL.

	Total Phosphorus	Orthophosphate (Reactive)
Replicate	Calculated TP Concentration [mg-P·L^{-1}]	Calculated OP Concentration [mg-P·L^{-1}]
1	0.0165	0.0120
2	0.0155	0.0120
3	0.0145	0.0100
4	0.0125	0.0100
5	0.0155	0.0140
6	0.0165	0.0150
7	0.0145	0.0130
8	0.0185	0.0110
9	0.0155	0.0100
Standard deviation	0.00166	0.00183
MDL = 2.896 * std. dev.	0.00480	0.00531
MRL = 3 * MDL	0.014	0.016

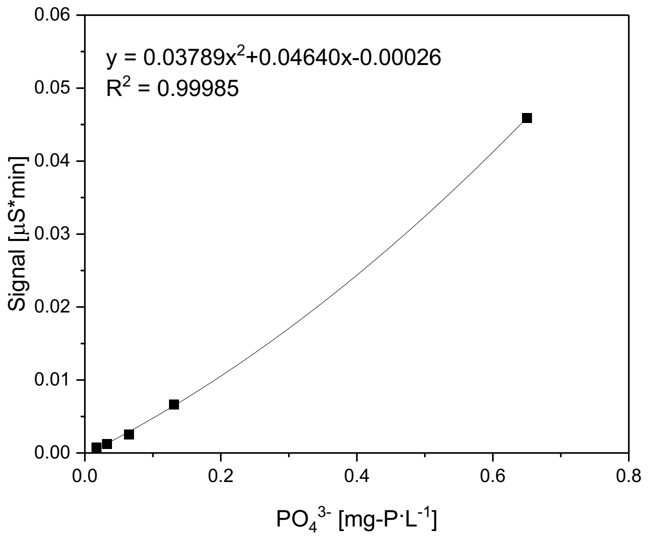

Figure S3. Calibration curve for PO_4^{3-} -P concentration (mg-P·L^{-1} as PO_4^{3-}) and peak area (µS·min) as measured on a Dionex ICS2100 anion ion chromatograph with a matrix background. Matrix was prepared from bench-scale PBR experiments, which were initiated with mix tank effluent from the full-scale system. Background matrix samples with no detectable orthophosphate (ICS200 signal of 0.00 µS·min) were acquired by filtering bench-scale PBR samples through 0.2 µm nitrocellulose filters after 300 minutes and 360 minutes of batch operation (orthophosphate was no longer detectable after 40 minutes of batch operation). Potassium orthophosphate was spiked into the background matrix samples to achieve final concentrations of 0.017, 0.032, 0.065, 0.13, and 0.65 mg-P·L^{-1} orthophosphate to generate the standard curve.

Table S3. PO_4^{3-} -P standard concentration (mg-P·L^{-1} as PO_4^{3-}), peak area (µS·min), retention time (min), calculated PO_4^{3-} -P concentration (mg-P·L^{-1} as PO_4^{3-}), standard deviation, MDL, and MRL for nine replicates each of the lowest four standards on the calibration curve in Figure S3 as measured on a Dionex ICS2100 anion ion chromatograph with a matrix background (as in Figure S3). From the lowest 4 points (0.017, 0.032, 0.065, and 0.13 mg-P·L^{-1} as PO_4^{3-}) on the calibration curve, the average method reporting limit (MRL) was 0.027 mg-P·L^{-1} as PO_4^{3-} and the MRL range was 0.022 to 0.037 mg-P·L^{-1} as PO_4^{3-}. The MRL was calculated as three times the method detection limit (MDL), which was the standard deviation of nine replicates (n = 9) multiplied by the t-value for n - 1 samples.

Standard PO_4^{3-} Concentration [mg-P·L^{-1}]	0.017	0.032	0.065	0.13
Replicate	Calculated PO_4^{3-} Concentration [mg-P·L^{-1}]	Calculated PO_4^{3-} Concentration [mg-P·L^{-1}]	Calculated PO_4^{3-} Concentration [mg-P·L^{-1}]	Calculated PO_4^{3-} Concentration [mg-P·L^{-1}]
1	0.0266	0.0307	0.0608	0.1386
2	0.0204	0.0348	0.0568	0.1386
3	0.0204	0.0307	0.0608	0.1333
4	0.0204	0.0368	0.0608	0.1386
5	0.0224	0.0307	0.0549	0.1386
6	0.0204	0.0307	0.0588	0.1333
7	0.0204	0.0307	0.0529	0.1298
8	0.0266	0.0307	0.0588	0.1280
9	0.0183	0.0286	0.0588	0.1316
Std. Dev.	0.00294	0.00253	0.00279	0.00424
MDL = 2.896*Std. Dev.	0.00851	0.00732	0.00807	0.01228
MRL = 3*MDL	0.026	0.022	0.024	0.037

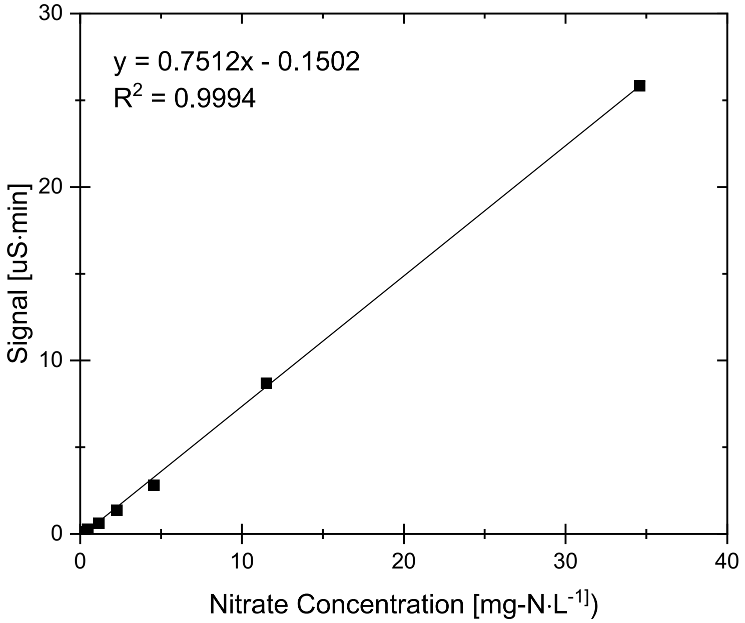

Figure S4. Calibration curve for NO_3^- -N concentration (mg-N·L^{-1} as NO_3^-) and peak area (μS·min) as measured on a Dionex ICS2100 anion ion chromatograph. Sodium nitrate at concentrations of 0.045, 0.11, 0.22, 0.45, 1.16, 2.27, 4,54, 11. 5, and 34.6 mg-N·L^{-1} nitrate were used to generate the standard curve.

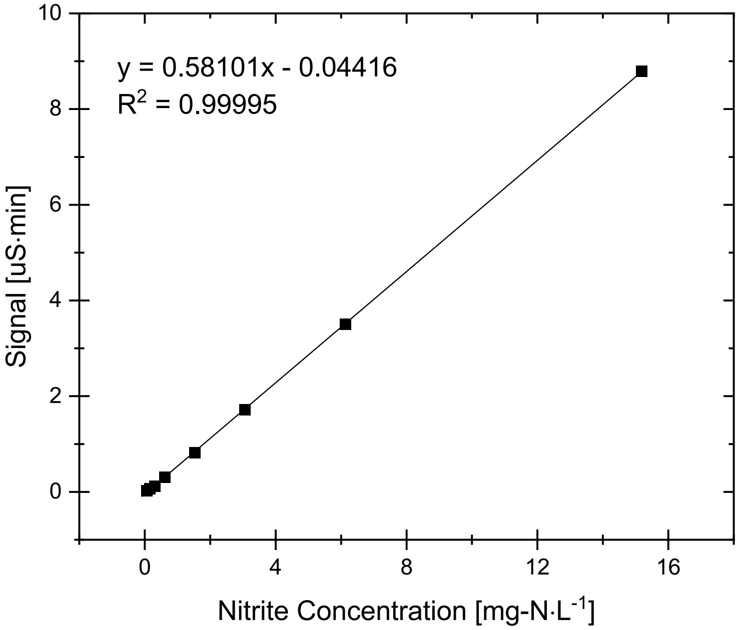

Figure S5. Calibration curve for NO_2^- -N concentration (mg-N·L^{-1} as NO_2^-) and peak area (μS·min) as measured on a Dionex ICS2100 anion ion chromatograph. Sodium nitrite at concentrations of 0.061, 0.15, 0.30, 0.61, 1.53, 3.06, 6.12, and 15.18 mg-N·L^{-1} nitrite were used to generate the standard curve.

S3. Characterization of long-term continuous performance

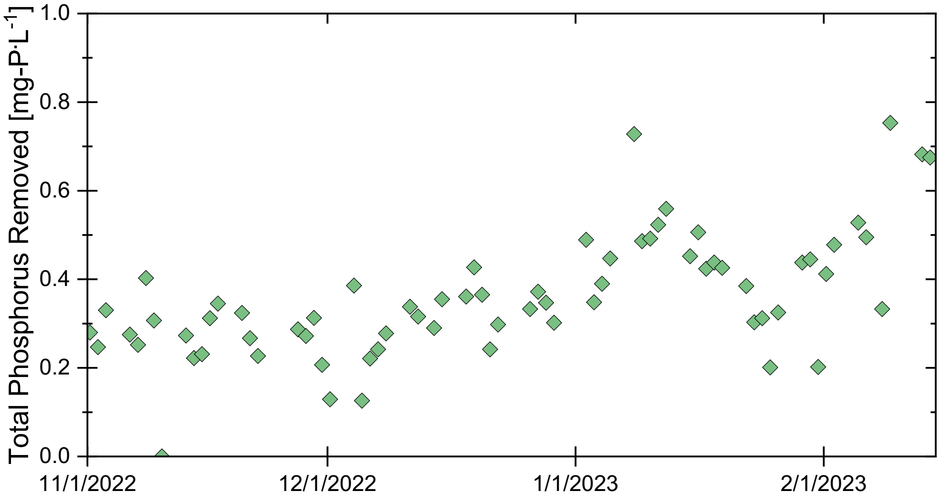

Figure S6. EcoRecover effluent total phosphorus removed (EcoRecover influent concentration minus effluent concentration) during a 3.5-month focus period, including a 3-month period of sustained excellent performance (<0.04 mg-P·L^{-1} TP).

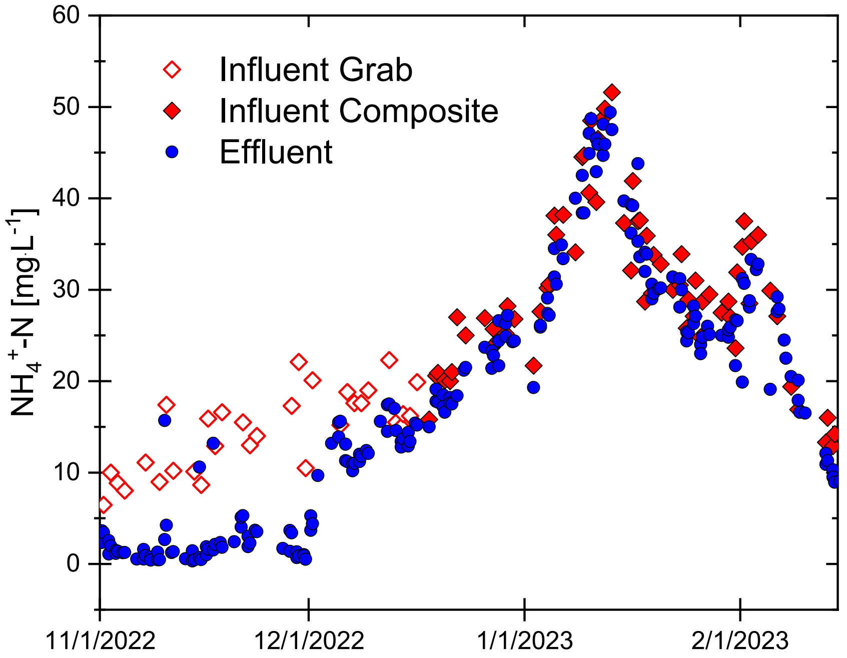

Figure S7. Ammonium (NH_4^+-N) concentration in influent (secondary effluent) and effluent for the period of November 1, 2022 to February 14, 2023 as measured on-site with Hach test kits TNT830, TNT831, and TNT832, depending on sample concentration. Influent NH_4^+-N was measured in 24-hour composite samples (filled diamonds), except during the period from Nov. 1, 2022 through Dec. 16, 2023 while supplemental NH_4^+-N was added to the secondary effluent and total influent NH_4^+-N was measured in grab samples (open diamonds). Effluent NH_4^+-N was measured in 24-hour composite samples.

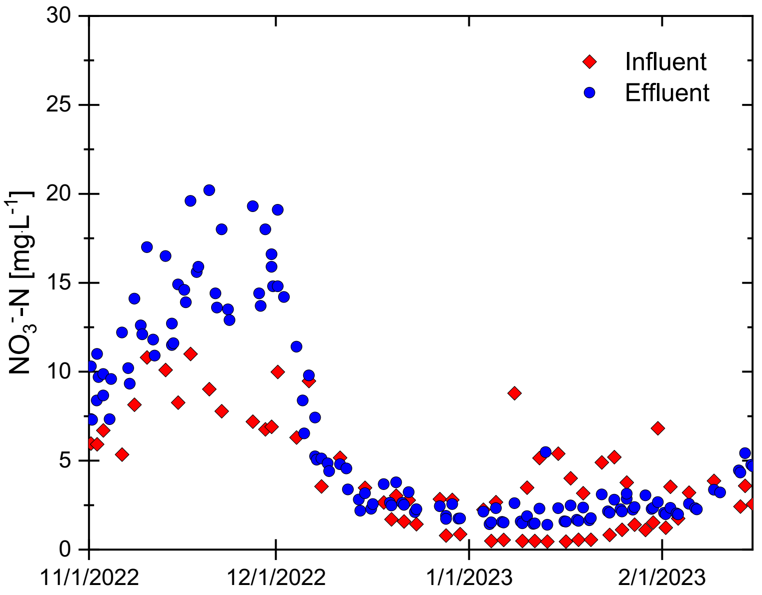

Figure S8. Nitrate (NH_4^+-N) concentration in influent (secondary effluent) and effluent grab samples for the period of November 1, 2022 to February 14, 2023 as measured on-site with Hach test kits TNT839 and TNT840, depending on sample concentration.

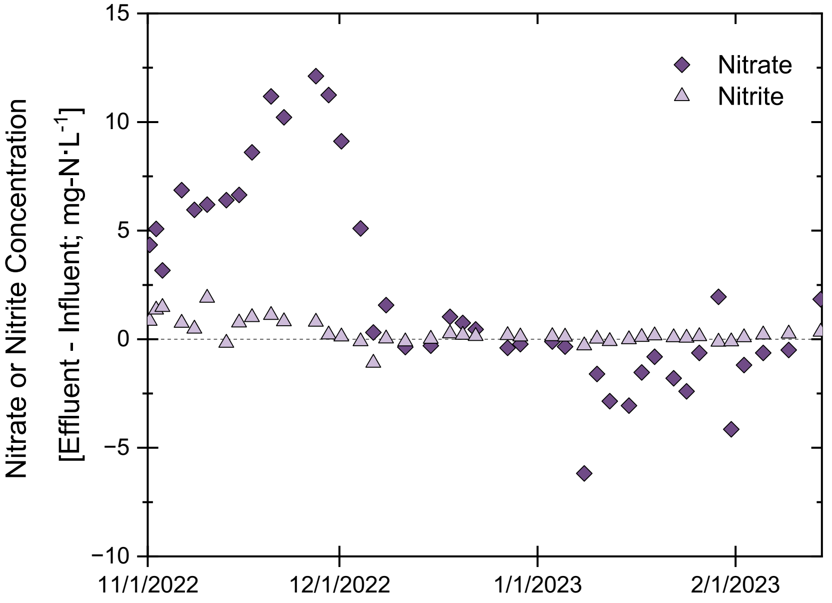

Figure S9. Difference in oxidized nitrogen species (NO_3^--N and NO_2^--N) concentrations in EcoRecover effluent and influent 24-h composite samples for the period of November 1, 2022 to February 14, 2023. Values above the zero indicate that the rate of increase in oxidized nitrogen species, due to nitrification, was greater than the consumption of oxidized nitrogen species by the mixed microalgal community. From December 8, 2022 to February 14, 2023, nitrification (and incomplete nitrification) was negligible; the difference between effluent and influent oxidized nitrogen species was -1.0 ± 1.8 mg-N·L^{-1} NO_3^- and 0.08 ± 0.15 mg-N·L^{-1} NO_2^-.

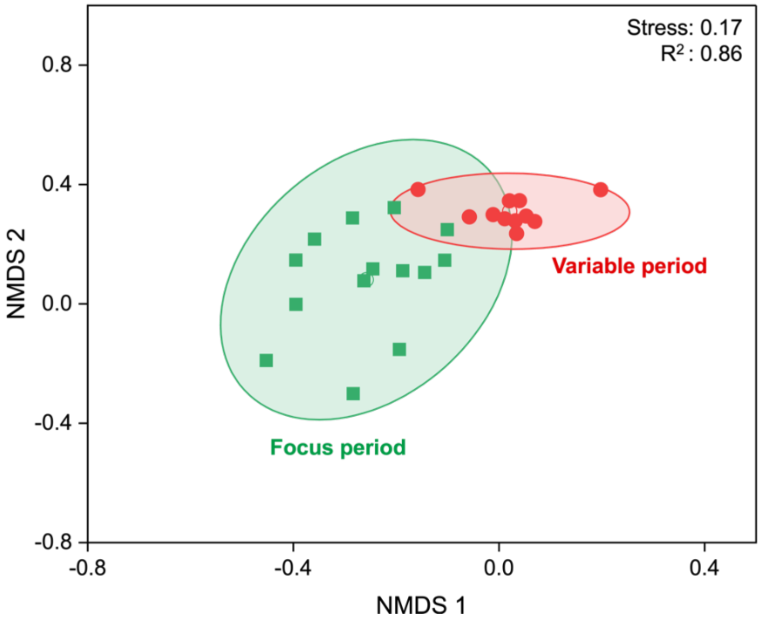

Figure S10. Non-metric multi-dimensional scaling (NMDS) plot showing the spatial distances among eukaryotic communities throughout the focus period (11/2/22 – 2/15/23) and a subsequent variable period (2/15/23 – 4/28/23), with ellipses containing 95% of cluster-assigned datapoints.

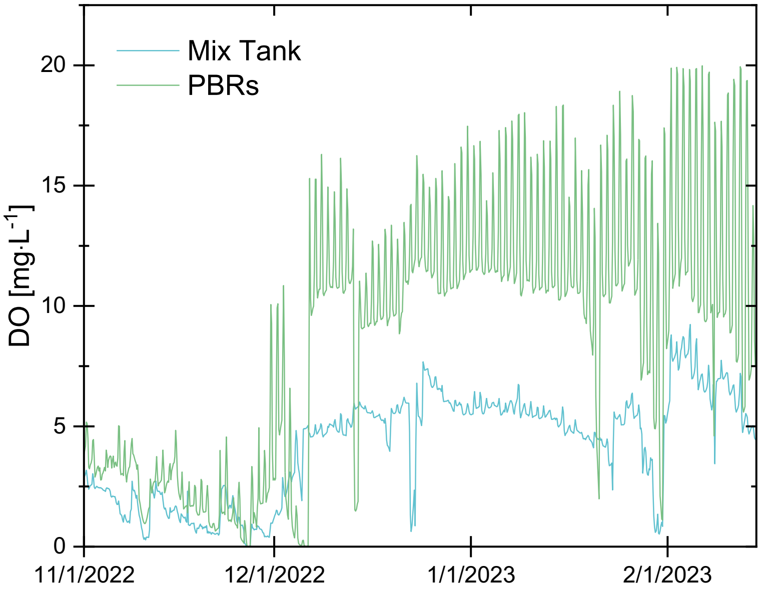

Figure S11. Dissolved oxygen (DO) concentration 180-minute average in the Mix tank effluent and PBR effluent for the period between November 1, 2022 to February 14, 2023 as measured by IQ SensorNet FDO Optical Dissolved Oxygen Probes (YSI Inc.).

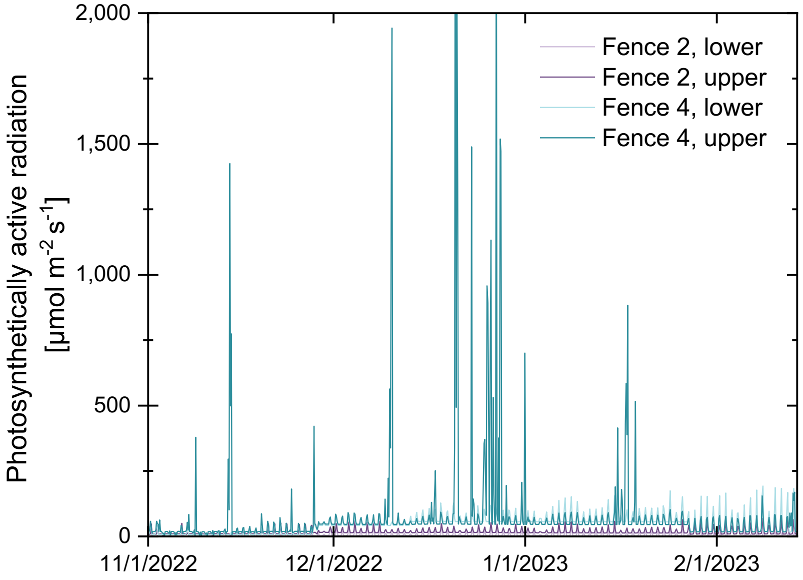

Figure S12. Photosynthetically active radiation (PAR) 180-minute average at four locations on the PBRs (two upper fences and two lower fences) for the period between November 1, 2022 to February 14, 2023. PAR was measured by Model SQ-214 PAR sensors (Apogee Inc.).

S4. Full-scale biomass composition trends

S4.1. Long-term continuous monitoring of solids phosphorus content

Elemental CHNP in freeze-dried biomass from the EcoRecover Mix tank and PBR culture were analyzed for samples beginning February 21, 2022 (Figure S12). Microalgae typically have 1% to 2% phosphorus; 80% and 75% of biomass samples from the Mix tank and PBRs, respectively, exceeded 2% phosphorus between February 21, 2022 to May 12, 2023. EcoRecover biomass phosphorus content ranged from 1.31% P (August 16, 2022; PBR) to 5.32% P (February 15, 2023, Mix tank) (Table S4).

Table S4. EcoRecover Mix tank and PBR biomass phosphorus content metrics from February 21, 2022 to May 12, 2023.

Phosphorus Content [%]	Mix tank	PBR
Minimum	1.50%	1.31%
Maximum	5.32%	5.08%
5th Percentile	1.72%	1.64%
95th Percentile	4.25%	4.03%
Median	2.85%	2.54%
Average	2.87%	2.70%

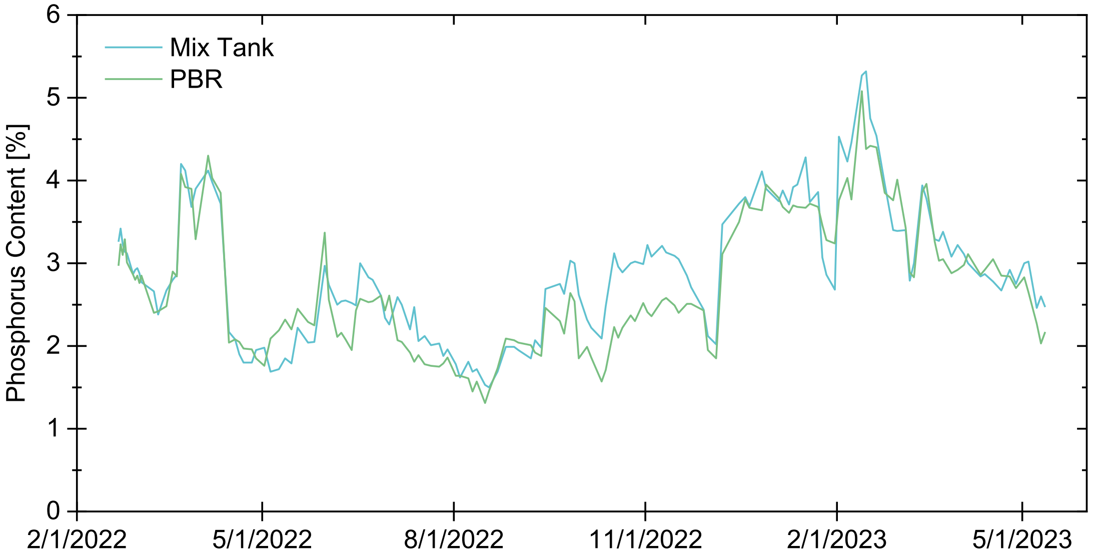

Figure S13. Solids phosphorus content in the EcoRecover Mix tank and PBR effluent mixed microalgal culture from February 21, 2022 to May 12, 2023. Biomass elemental phosphorus was quantified in duplicate lyophilized samples through ICP-MS.

S4.2. Sample selection for SEM-EDS

To understand the potential phosphorus removal mechanisms across the range of EcoRecover operating conditions and system solids phosphorus contents, a total of 12 samples were analyzed via SEM-EDS. Samples for SEM-EDS analysis were selected to represent extremes in biomass %P across the entire sampling period, biomass from periods with and without coagulant use, and start and end points in batch experiments (Table S5).

Table S5. Criteria for sample selection for SEM-EDS analysis and key system operating conditions and performance during the sampling date.

Criteria	Overall biomass %P	Sample Date	Performance	Influent pH	Mix tank pH	PBR pH	Permeate pH	DO diel rhythm?	SRT (d)	Chemical dosing?	How long with/without chemical?
**Greatest %P**	5.08%	2/13/2023	Good, but very end of excellent performance.	7.2-7.5	7.2	7.0	7.2-7.3	Yes	Not reported, 5.6 d on 2/14/23	Yes, PAC (polyaluminum chloride)	~7 months
**Least %P**	1.31%	8/16/2022	Tail end of July-Aug '22 upset, SBR changes upstream (6/1/22)	7.2	7.3-7.5	7.0	7.2-7.4	Yes	3.4	Yes, Alum (aluminum sulfate)	1+ month with Alum, just under 2 months since PAC
**Longest period without coagulant**	1.95%	6/13/2022	Beginning of July-August '22 upset, SBR changes upstream (6/1/22)	7.4	7.2-7.3	7.0	7.3-7.4	Poor	4.1	No	1 month, PAC ended ~5/18/22
**2nd Longest without coagulant**	2.08%	6/10/2022	Beginning of July-August '22 upset, SBR changes upstream (6/1/22)	7.5	7.4	7.1	7.5	Good	4.6	No	3 weeks
**2nd Least time without coagulant**	2.56%	6/2/2022	Approaching upset but barely elevated effluent OP, immediately post-SBR changes	7.5	7.1	7.1	7.2	Good	5.3	No	2 weeks
**Least time without coagulant**	2.29%	5/23/2022	Good, pre-SBR changes	7.5	7.1	Not reported	7.2	Good	4.1	No	<1 week
**PBR Replicate 1 t = 0**	3.96%	5/13/2022	Good	Batch experiments controlled at 6.8-7.2				Good	4	Yes, PAC	4 months
**PBR Replicate 1, t = 360 min**	2.82%										
**Mix tank Replicate 1, t = 240 min**	3.79%										
**Diatom spike in omics data**	1.81%	7/13/2022	On recovery side of significant upset caused by upstream SBR changes	7.2	7.2	6.9	7.3	Dampened but cycling	3.4	Yes, Alum	1 week
**Good performance**	4.30%	4/5/2022	Good					Good		Yes, PAC	2 months
**Good performance**	1.96%	4/26/2022	Good					Good		Yes, PAC	3 months

During periods of coagulant use, phosphorus measured on the solids surface was generally less than that measured on solids during periods without coagulant use (Table S6). Granules, enriched with phosphorus and metals, were only present in the solids samples during periods of coagulant use or immediately after cessation of coagulant use (Figure S13).

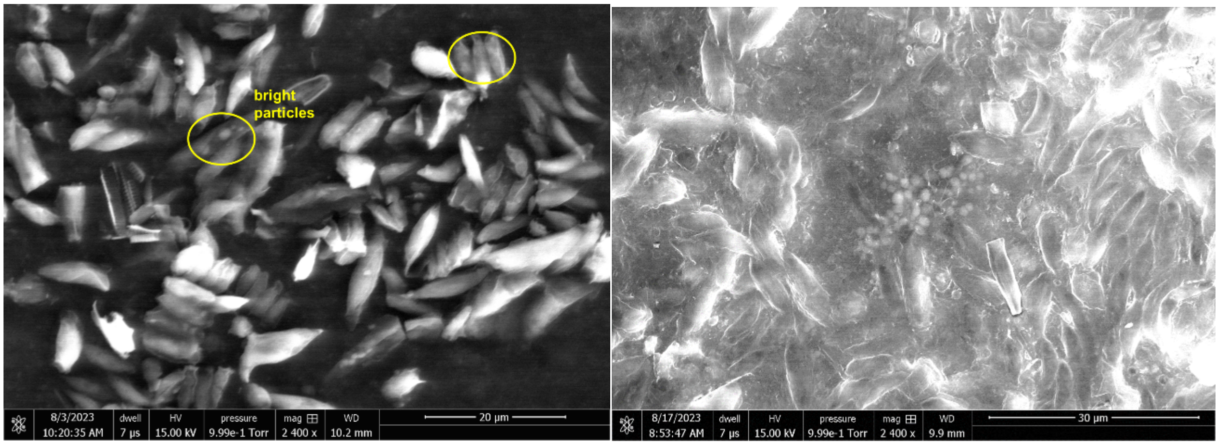

Figure S14. SEM-EDS images of lyophilized and ground EcoRecover PBR solids from February 13, 2023 (left) and April 5, 2022 (right), during a period of coagulant use. Bright particles represent highly localized elevated concentrations of metals and phosphorus. The composition of the granules within the EPS could not be determined due to interference from the carbon spectra but had elevated levels of metals and P.

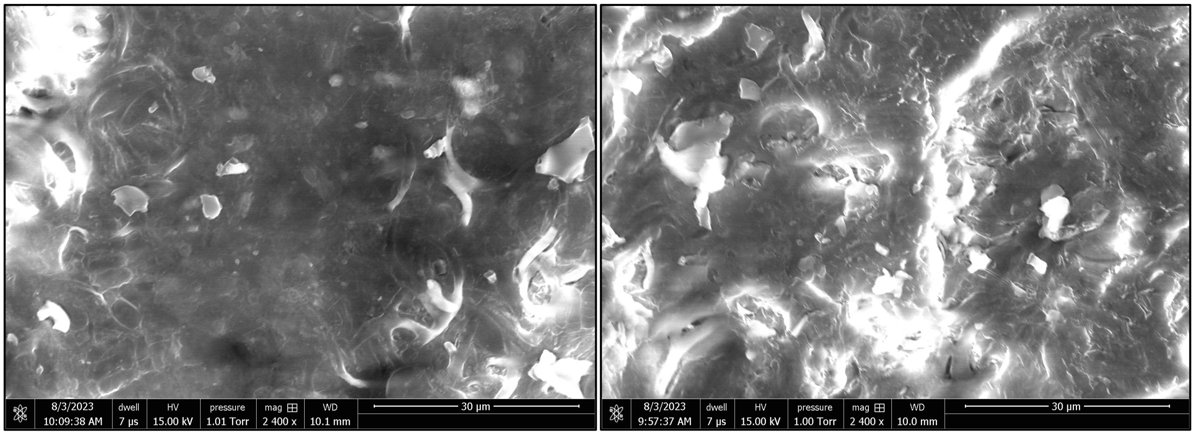

Figure S15. SEM-EDS images of lyophilized and ground EcoRecover PBR solids from June 10, 2022 (left) and June 13, 2023 (right), during 4-week period without coagulant use. No metal- or P-rich precipitate was observed.

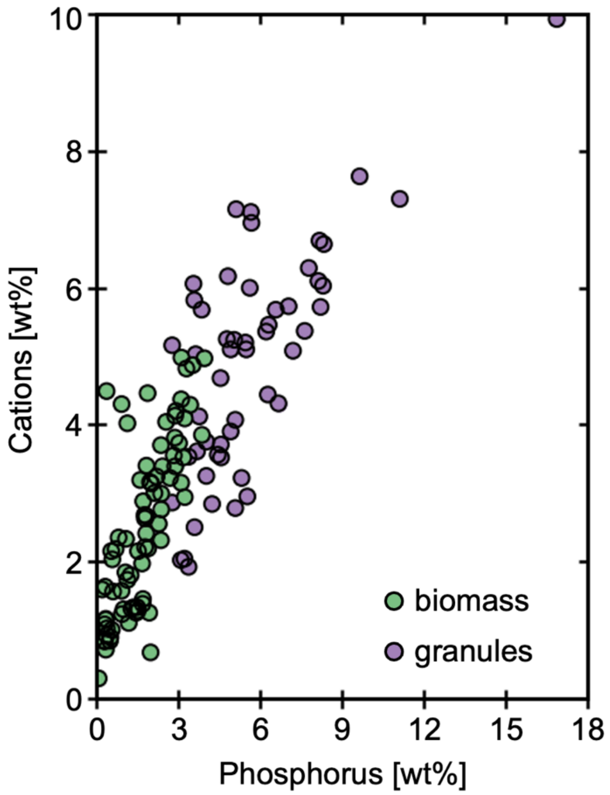

Figure S16. Phosphorus and metals (sum of Na, Mg, Al, K, Ca) weight percentages of analyzed granules (purple) and biomass without bright particles (green) based on SEM-EDS. The exact composition of the granules could not be determined due to interference from the carbon spectra, but granules had elevated levels of phosphorus and metals relative to the rest of the analyzed biomass.

S4.3. Long-term continuous monitoring of solids N:P ratio

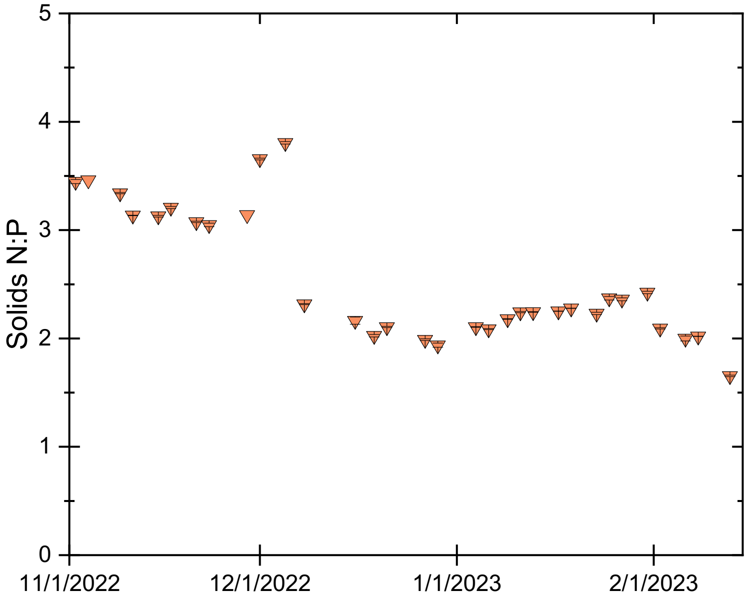

Figure S17. Solids N:P mass ratio for the period between November 1, 2022 to February 14, 2023. Error bars represent minimum and maximum values from duplicate CHN analyses; phosphorus analyses were done in single samples.

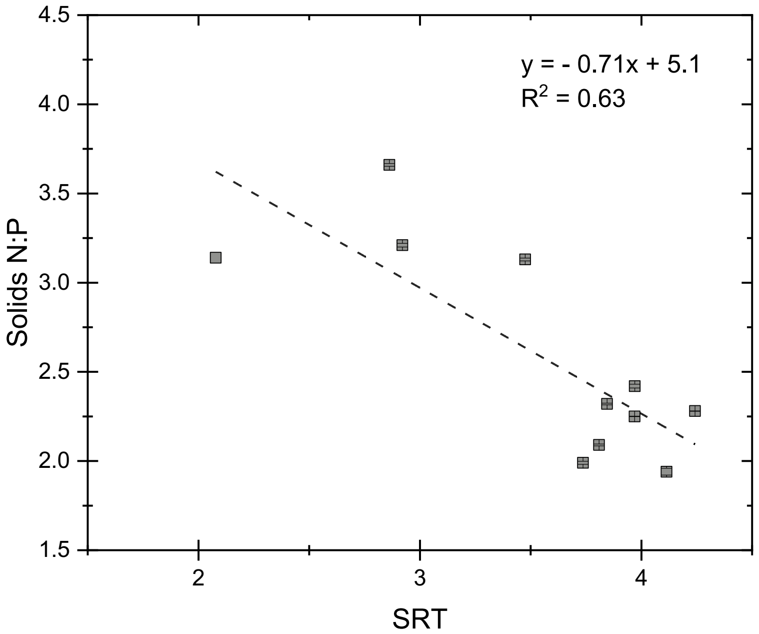

Figure S18. The EcoRecover solids N:P mass ratio had a negative linear correlation with the system SRT (slope: -0.71, y-intercept: 5.1, $R^2 = 0.63$) during a 3-month period of excellent performance (November 15, 2022 to February 14, 2023). Error bars represent minimum and maximum values from duplicate CHN analyses; phosphorus analyses were done in single samples.

S5. Flow imaging microscopy

To prepare aqueous samples for processing, 1 mL aliquots were diluted in deionized water by a factor of 4-6x. Dilution rate was estimated based on the previous day's measurements and by visual inspection. Diluted samples were loaded into the intake syringe port of a FlowCam 5000 flow imaging microscope (Yokogawa Fluid Imaging Technologies, Inc.). In FlowCam's VisualSpreadsheet 5 software, runs were configured to process 0.5 milliliters of sample volume. Once a run was completed, background objects that were not automatically ignored during sample capture were manually removed. Background objects refer to particles that appear in every digital image frame due to adhesion to the walls of the flow chamber, and thus appear as thousands of identical duplicates in sample results. Duplicates were identified as particles of similar appearance with identical or similar x-y coordinates. Out of focus particles were then removed from the run data with a feature-based filter. A threshold cutoff was set for the edge-gradient and transparency particle features to distinguish out of focus particles. After background and out of focus particle screening, run data was saved to disk.

For aggregate time series analysis, particle feature data were provided as input to a neural network-based classification model. The classification model generated per-particle taxonomic class predictions, which were aggregated to estimate daily total abundance of microalgal populations. To develop this classification model, training datasets were first manually annotated into image libraries corresponding to each of the dominant taxonomic groups identified across multiple sample points. Each class consisted of 4,000 to 8,000 representative particle images. Using the deep learning library TensorFlow (version 2.5.0, Python 3.9, Apache License 2.0), a bespoke neural network was created from randomized starting weights. The model's architecture consisted of: an input layer sized to the total width of the FlowCam-exported particle feature data tables, a series of fully connected hidden layers, and an output layer whose size corresponded to the number of taxonomic classes to be predicted. Training data class sizes were increased, and model architectures and hyperparameters were adjusted until training yielded a classification model achieving > 90% overall accuracy with inter-class accuracy variability less than 10% (i.e., all classes between 85% and 95% accuracy). The model architecture and weights of this model were saved to disk and the same model was used to predict taxonomic identities of all particles for the full time series.

S6. Susceptibility to process upsets

Periods of process upset were characterized by prolonged exceedance of the 0.04 $mg\text{-}P\cdot L^{-1}$ permit.

S6.1. Upstream treatment process changes and altered EcoRecover influent quality:

On June 7, 2022, modifications were made to the SBRs upstream of the EcoRecover system that rapidly increased the TP and NO_3^- and decreased the NH_4^+ EcoRecover system influent concentrations. The system biomass concentrations (300 to 400 $mg\cdot L^{-1}$ TSS) were insufficient to treat the high SBR effluent nutrient concentrations; without sufficient treatment capacity, the EcoRecover system effluent TP exceeded 0.04 $mg\text{-}P\cdot L^{-1}$ and dramatically increased beginning on June 7, 2022, to peak on July 1 and 2, 2022 at 4.4 $mg\text{-}P\cdot L^{-1}$ until concentrations below 0.04 $mg\text{-}P\cdot L^{-1}$ were again achieved on July 30, 2022. During the upset, flow was rerouted to bypass the EcoRecover system. 75% reduction in flow through the PBRs in a greenhouse in July caused the culture temperatures to reach 90º to 95° F on July 9 and 10, 2022, further delaying performance recovery (SI Figure S18A, unhealthy culture). Full flow was restored to the EcoRecover system on July 11, 2022. With gradual increase in alum addition to the WWTF headworks, EcoRecover influent TP concentrations gradually decreased to manageable levels (<1 $mg\cdot L^{-1}$) by the week of July 25, 2022 (SI Figure S18B, healthy culture).

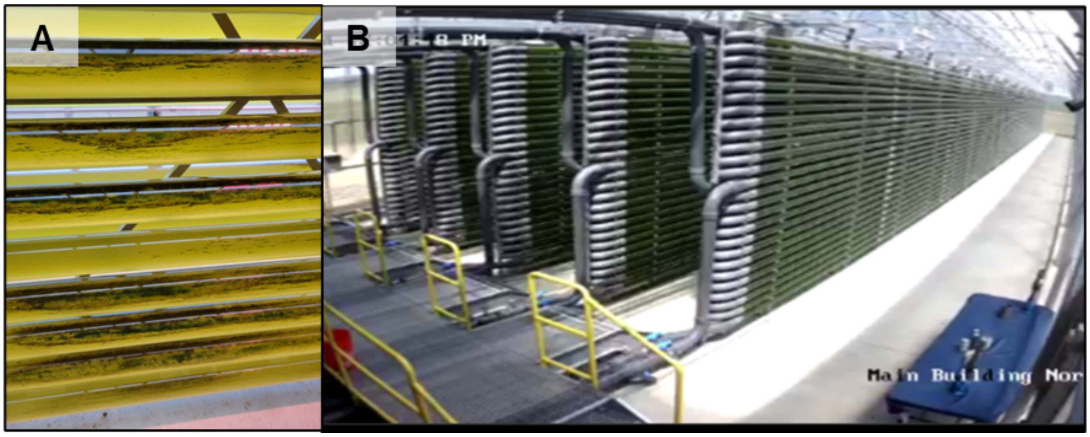

Figure S19. (A) EcoRecover PBR fence from the week of July 11, 2022 after system upset and high temperature exposure; stressed culture with poor phosphorus removal flocculates and attaches to the glass. (B) EcoRecover PBR fences from on-site greenhouse camera on July 20, 2022 showing greener, denser culture.

S6.2. Quaternary ammonium inhibition:

Process upsets alerted the EcoRecover operators to a potential inhibitory compound in the influent. Beginning on January 26, 2022, an external laboratory, Badger Laboratories Inc. (Wisconsin, USA), quantified a panel of possible inhibitory compounds in the EcoRecover influent, including quaternary ammonium (QA). QA concentrations were unusually high, up to 2.5 ppm. Quantification of QA continued on site; between January 26, 2022 and July 7, 2023, the average EcoRecover influent QA concentration was 0.7 ± 0.2 ppm (Quartile 1: 0.5 ppm, Quartile 2: 0.6 ppm, Quartile 3: 0.8 ppm, Quartile 4: 2.5 ppm). Concentrations of eleven QA compounds have been reported to be inhibitory (EC_{50}) to *C. vulgaris* between 0.11 to 0.20 ppm,[1–3] and 0.96 to 2.63 ppm dodecylethyldimethyl-ammonium bromide (DEAB) was reported as inhibitory to *S. intermedius* and *D. chlorelloides*.[4] Culture sensitivity to QA is a challenge to the EcoRecover system performance that has been addressed by adding QuatKill (ChemTreat, Inc., Virginia, USA), a solution designed to neutralize cationic biocides, to the influent.

S7. Bench-scale batch experiments

In the PBR batch experiments, 30.39 ± 0.13 mg-N·L^{-1} NH_4^+ was removed, with some removal due to nitrification which increased the concentrations of nitrate by 6.8 ± 1.5 mg-N·L^{-1} and nitrite by 0.19 ± 0.07 mg-N·L^{-1}. In the Mix tank experiments, 4.7 ± 0.1 mg-N·L^{-1} NH_4^+ was removed and nitrification increased the concentrations of nitrate and nitrite by 3.0 ± 0.5 mg-N·L^{-1} and 1.2 ± 0.4 mg-N·L^{-1}, respectively, which demonstrates NH_4^+ removal almost solely through nitrification. In the PBR batch experiments, 23% of the initial NH_4^+ was removed through nitrification and the remaining majority was removed through biomass assimilation.

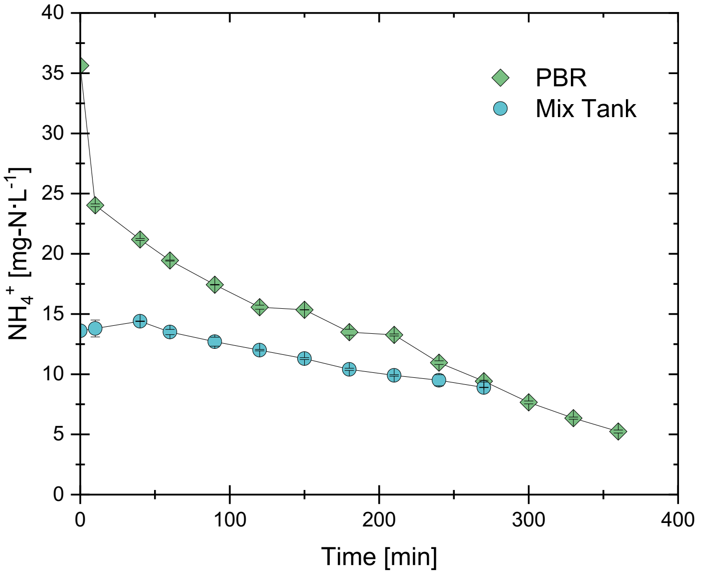

Figure S20. Average NH_4^+-N removal over time in batch, bench-scale mix tank and PBR duplicate experiments. Error bars represent minimum and maximum values from duplicate analyses.

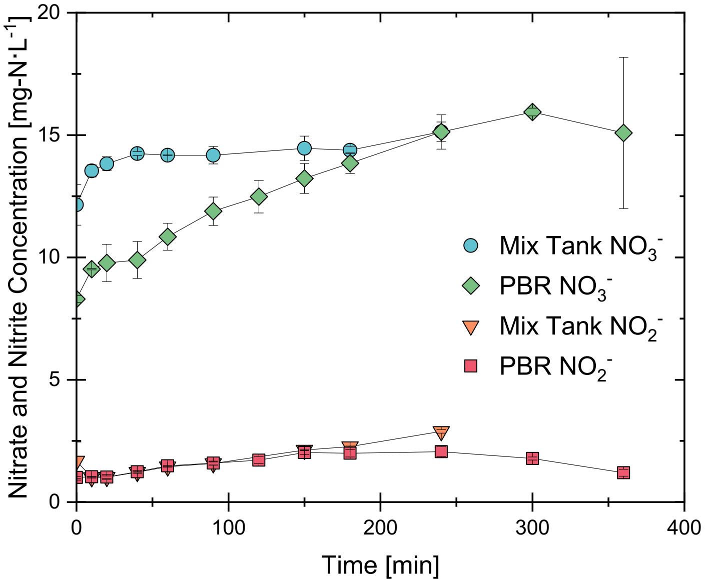

Figure S21. Average NO_3^--N and NO_2^--N concentrations over time in batch, bench-scale mix tank and PBR duplicate experiments. Error bars represent minimum and maximum values from duplicate analyses.

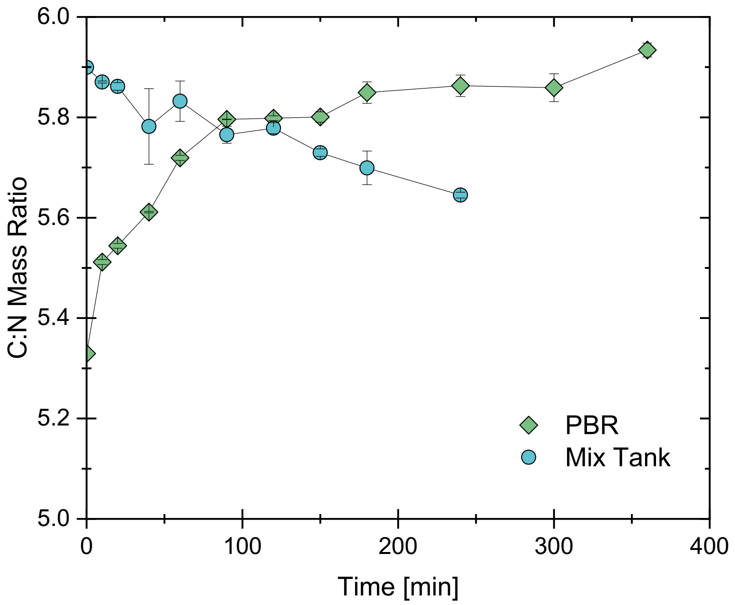

Figure S22. Carbon:nitrogen (C:N) mass ratio for bench-scale experiments that replicated the conditions of the mix tank and PBRs. Error bars represent minimum and maximum values from duplicate analyses.

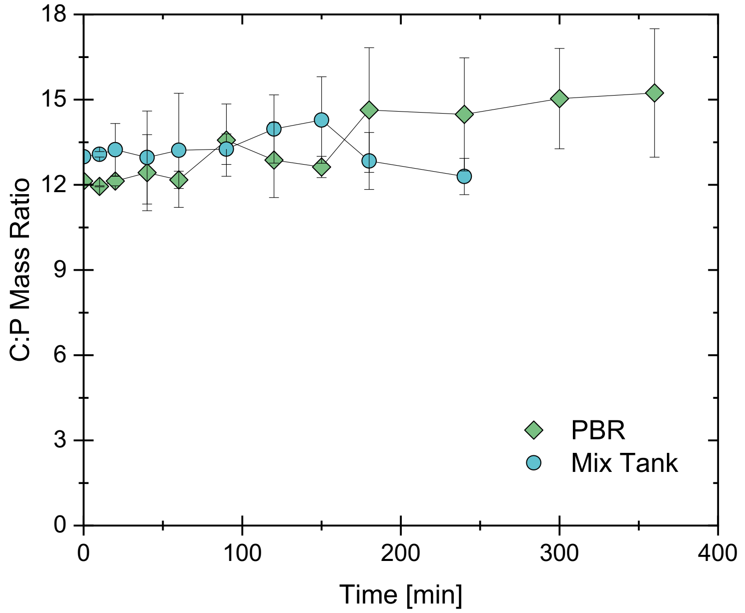

Figure S23. Carbon:phosphorus (C:P) mass ratio for bench-scale experiments that replicated the conditions of the mix tank and PBRs. Error bars represent minimum and maximum values from duplicate analyses.

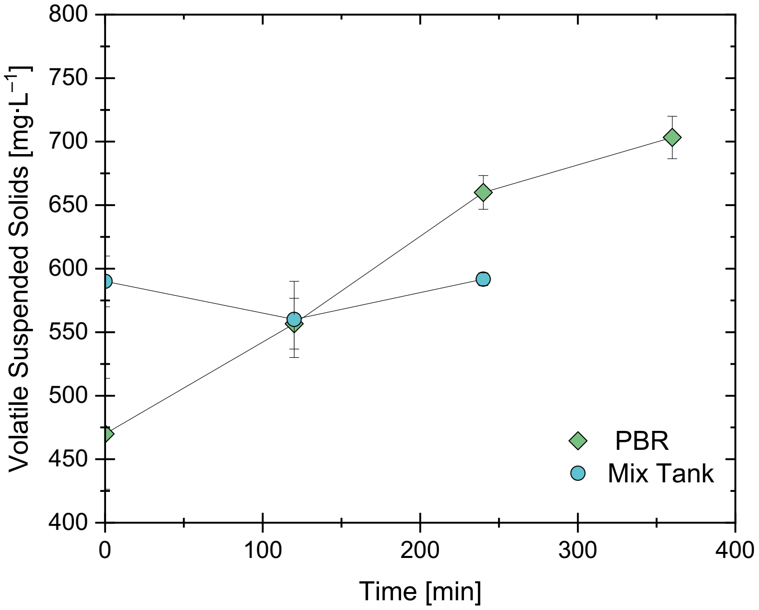

Figure S24. Volatile suspended solids (VSS) concentration for bench-scale experiments that replicated the conditions of the mix tank and PBRs. Error bars represent standard deviation from triplicate analyses.

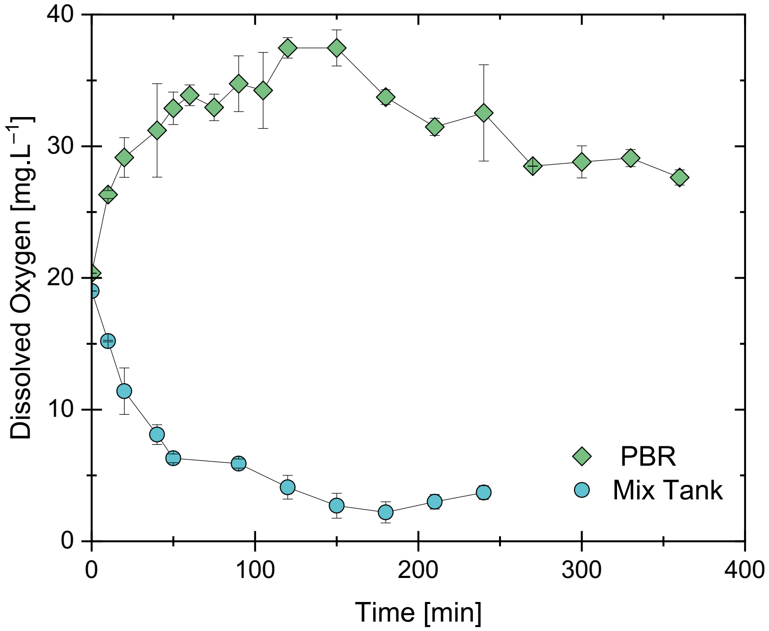

Figure S25. Dissolved oxygen concentrations over time for bench-scale experiments that replicated the conditions of the mix tank and PBRs. Error bars represent minimum and maximum values from duplicate analyses.

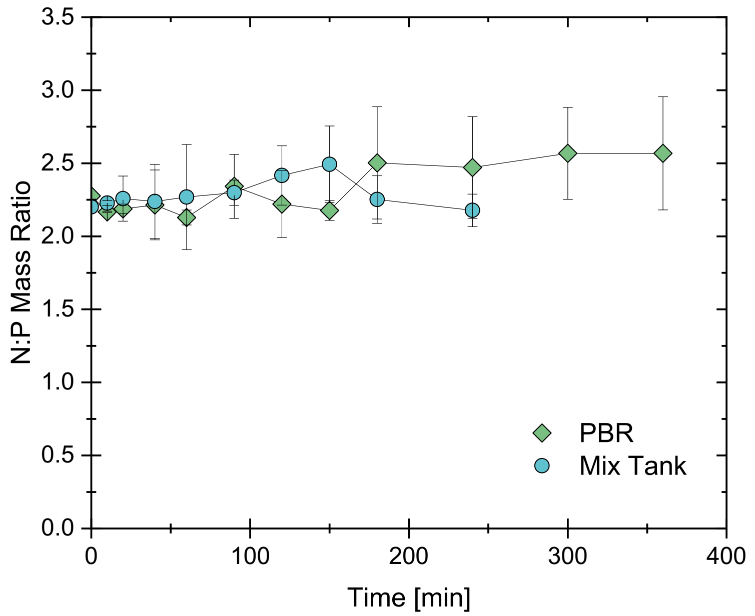

Figure S26. Nitrogen:phosphorus (N:P) mass ratio for bench-scale experiments that replicated the conditions of the mix tank and PBRs. Error bars represent minimum and maximum values from duplicate analyses.

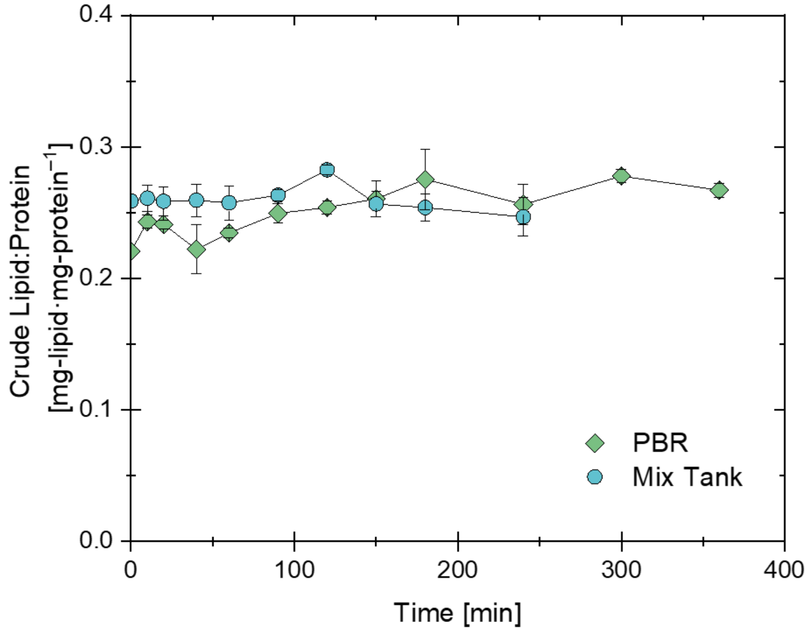

Figure S27. Crude lipid to protein mass ratio for bench-scale experiments that replicated the conditions of the mix tank and PBRs. No significant change in biomass lipids content was demonstrated over the course of the experiments. Error bars represent minimum and maximum values from duplicate analyses.

S8. Full-scale biomass composition trends

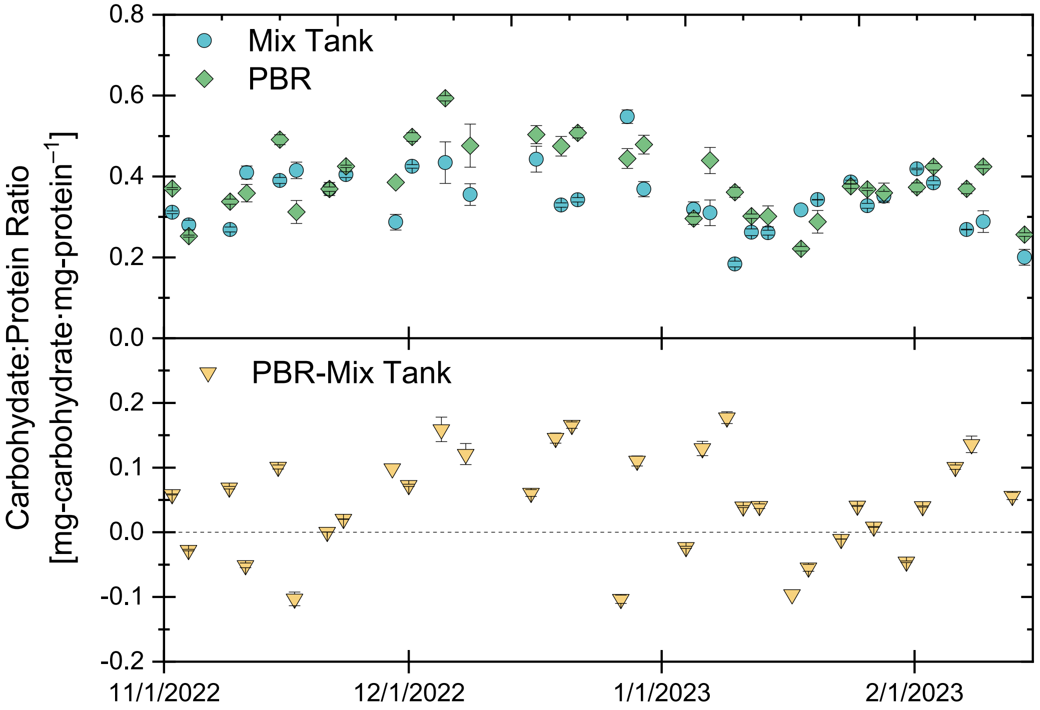

Figure S28. Top: Biomass carbohydrate:protein ratio (by mass) in the full-scale Mix tank and PBR effluent between November 1, 2022 to February 14, 2023. Error bars represent minimum and maximum values from duplicate analyses. Bottom: Difference between the PBR and mix tank biomass carbohydrate:protein ratio between November 1, 2022 to February 14, 2023. Error bars represent the propagated error (Equation S1) of duplicate analyses.

Equation S1. Propagation of error for subtraction of Mix tank and PBR duplicate analyses

$$C \pm c = (A - B) \pm \sqrt{(a)^2 + (b)^2}$$

where,

C is the average value of the Mix tank C:N ratio subtracted from that of the PBR average value

c is ± the propagated relative error

A is the average PBR C:N ratio

a is the relative error of the duplicate PBR C:N measurements

B is the average Mix tank C:N ratio

b is the relative error of the duplicate PBR C:N measurements
